# The extracellular juncture domains in the intimin passenger adopt a constitutively extended conformation inducing restraints to its sphere of action

**DOI:** 10.1038/s41598-020-77706-7

**Published:** 2020-12-04

**Authors:** Julia Weikum, Alina Kulakova, Giulio Tesei, Shogo Yoshimoto, Line Vejby Jægerum, Monika Schütz, Katsutoshi Hori, Marie Skepö, Pernille Harris, Jack C. Leo, J. Preben Morth

**Affiliations:** 1 Membrane Transport Group, Centre for Molecular Medicine Norway (NCMM), Nordic EMBL Partnership, University of Oslo, P.O. Box 1137, Blindern, 0318 Oslo, Norway; 2 Enzyme and Protein Chemistry, Section for Protein Chemistry and Enzyme Technology, Department of Biotechnology and Biomedicine, Technical University of Denmark, Søltofts Plads, 2800 Kgs. Lyngby, Denmark; 3 Department of Chemistry, Technical University of Denmark, Kemitorvet Building 207, 2800 Kgs. Lyngby, Denmark; 4 Department of Chemistry, University of Copenhagen, Universitetsparken 5, 2100 Copenhagen, Denmark; 5 Structural Biology and NMR Laboratory and The Linderstrøm-Lang Centre for Protein Science, Department of Biology, University of Copenhagen, Copenhagen N, Denmark; 6 Department of Biomolecular Engineering, Graduate School of Engineering, Nagoya University, Furo-cho, Chikusa-ku, Nagoya, 464-8603 Japan; 7 Interfaculty Institute for Microbiology and Infection Medicine, Institute for Medical Microbiology and Hygiene, University Hospital Tübingen, 72076 Tübingen, Germany; 8 Division of Theoretical Chemistry, Department of Chemistry, Lund University, 221 00 Lund, Sweden; 9 Department of Biosciences, University of Oslo, P.O. Box 1066, Blindern, 0316 Oslo, Norway; 10 Department of Biosciences, Nottingham Trent University, Nottingham, NG11 8NS UK; 11 Institute for Experimental Medical Research (IEMR), Oslo University Hospital, Ullevål, PB 4956, Nydalen, 0424 Oslo, Norway

**Keywords:** Structural biology, Atomic force microscopy, Molecular modelling, SAXS, X-ray crystallography, Bacterial structural biology, Pathogens, Biochemistry, Biophysics, Structural biology, Mathematics and computing

## Abstract

Enterohemorrhagic and enteropathogenic *Escherichia coli* are among the most important food-borne pathogens, posing a global health threat. The virulence factor intimin is essential for the attachment of pathogenic *E. coli* to the intestinal host cell. Intimin consists of four extracellular bacterial immunoglobulin-like (Big) domains, D00–D2, extending into the fifth lectin subdomain (D3) that binds to the Tir-receptor on the host cell. Here, we present the crystal structures of the elusive D00–D0 domains at 1.5 Å and D0–D1 at 1.8 Å resolution, which confirms that the passenger of intimin has five distinct domains. We describe that D00–D0 exhibits a higher degree of rigidity and D00 likely functions as a juncture domain at the outer membrane-extracellular medium interface. We conclude that D00 is a unique Big domain with a specific topology likely found in a broad range of other inverse autotransporters. The accumulated data allows us to model the complete passenger of intimin and propose functionality to the Big domains, D00–D0–D1, extending directly from the membrane.

## Introduction

The gastrointestinal pathogens, enterohemorrhagic *Escherichia coli* (EHEC) and enteropathogenic *Escherichia coli* (EPEC) pose a severe public health threat^1^. EHEC is a food-borne human pathogen leading to both sporadic infections and epidemics, in particular outbreaks of hemorrhagic colitis (bloody diarrhea) and hemolytic uremic syndrome^2,3^. The largest outbreak occurred in Germany 2011 with 4000 cases of EHEC gastroenteritis and more than 850 reported cases of hemolytic uremic syndrome cases, leading to the death of 54 people^3–5^. EPEC is a major contributor to diarrhea in low-income countries, being a leading cause of child mortality^6^. EHEC and EPEC are highly related and share many virulence determinants and features, many of which are encoded on a pathogenicity island named the locus of enterocyte effacement (LEE)^7,8^. Among them, an essential virulence factor for adherence to the host cells is the outer membrane protein and virulence factor intimin, the gene product of the *eaeA* locus^9^. In addition to EHEC and EPEC, intimin variants are found in several other attaching and effacing pathogens, such as *Citrobacter rodentium* and *Hafnia alvei*^10^.

Intimin is a 94 kDa outer membrane protein, essential for the intimate attachment of bacterial cells to the host cell surface, followed by actin pedestal formation^8^. Before intimin binding, the translocated intimin receptor (Tir) is injected into the host cell by the bacterial type 3 secretion system and subsequently inserted into the host plasma membrane to promote the intimate attachment^11,12^. Intimin is classified as an inverse autotransporter, a subclass of the type 5 secretion system that transports the C-terminal extracellular region or passenger through the lumen of the N-terminal β-barrel located in the outer membrane^13^. In the following, we will refer to the extracellular region only as the passenger, as recommended by Drobnak et al*.*^14^. The secretion of the passenger proceeds through a hairpin-like intermediate, i.e. the membrane-proximal (N-terminal) part of the passenger is exported first, followed by the rest of the passenger with the C-terminus reaching the surface last^15^. Sequential folding of the individual subdomains is the main driving force for the secretion process^16^.

The intimin passenger forms a rod-like extension consisting of four tandem bacterial immunoglobulin-like (Big) domains (subdomains D00–D0–D1–D2) capped by a C-terminal C-type lectin-like domain (D3). This architecture is similar to other inverse autotransporters, such as *Yersinia pseudotuberculosis* invasin^17^. The C-type lectin-like domain (D3) at the C-terminus forms a superdomain with D2, which combined create the functional Tir-binding region, described earlier in complex with the binding domain of Tir (PDB: 1F02)^18^. Although bioinformatic and biophysical evidence suggested that subdomains D00 and D0 are also Ig-like domains^16,18^, no structural information has been available for these domains. Especially the structure of D00 and its connection to the neighboring subdomains have been discussed as D00 is located at the juncture between the outer membrane-bound β-barrel and the remaining extracellular passenger subdomains^19^. D00 represents the first subdomain upon excretion, and its folding was hypothesized to provide the driving force for extracting the remaining Big-domain in the intimin passenger^16,19^.

Here, we describe the crystal structures of the combined intimin domains D00–D0 and D0–D1 from the EPEC strain E2348/69 determined at 1.5 Å and 1.8 Å resolution, respectively. These structures represent the last missing structural information for the complete passenger of intimin. We show that the D00 structure represents a Big domain. However, in comparison with D0 and D1, it exhibits higher structural resemblance to the general Ig folds and remarkable topological resemblance with the mammalian cadherins. The D0 domain shows, as expected, a high structural similarity with the D1 intimin domain. The crystal structures in combination with small-angle X-ray scattering (SAXS), and in silico simulation data, suggest that D00–D0 adopts a more permanently extended and rigid conformation, likely stabilized by a short connector region between the D00 and D0 domains. The D0–D1 construct adopts a mostly bent conformation and is expected to be more dynamic as predicted by simulation studies. The structural integrity of intimin is independent of calcium and thus differs from specific adhesins and cadherins that commonly are regulated and rigidified upon calcium binding^20,21^. We hypothesize that the constitutively extended conformation of D00–D0 will increase the radius of probable interaction between the passenger and the bacterial Tir receptor on the host cell plasma membrane, positioning the Tir-binding region further away from the surface of the outer membrane. This would allow a faster binding to Tir following secretion of intimin to the bacterial surface.

## Results

### Crystal structure of the *E. coli* intimin subdomains D00–D0 exhibits an elongated conformation while D0–D1 shows a bent structure

**Figure 1:**
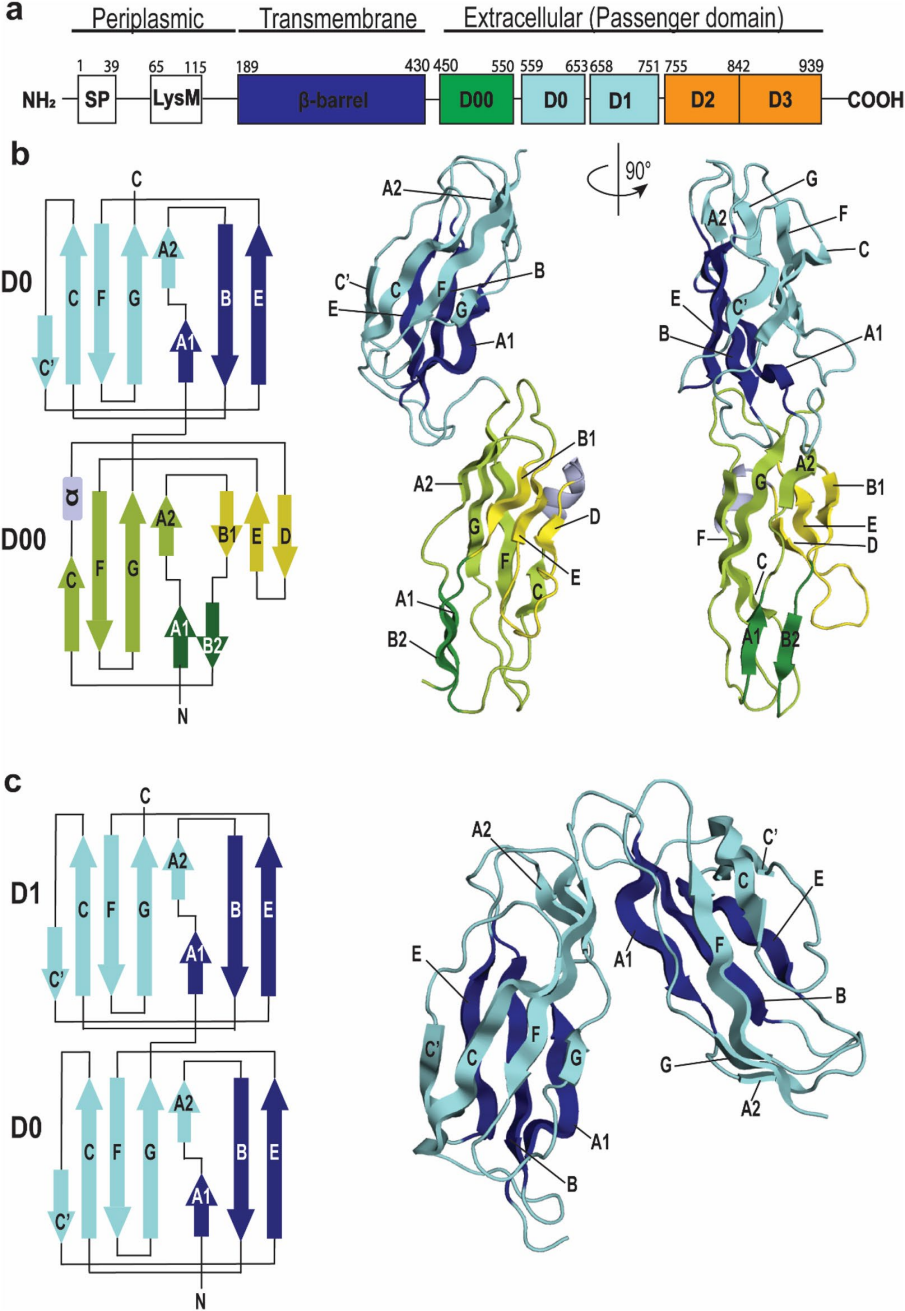
Crystal structure of the *E. coli* intimin subdomains D00–D0 exhibits an elongated conformation while D0–D1 shows a bent structure. (**a**) Domain architecture of *E. coli* intimin is represented based on the PDB structures exhibiting the periplasmic domain (PDB: 2MPW), including a signal peptide (SP), transmembrane β-barrel (PDB: 4E1S) and extracellular domains (PDB: 1F02). (**b**) Topology and ribbon diagram of D00–D0. D0 shows a classical bacterial Ig-like domain 1 (Big1) motif consisting of a β-sandwich, opposing antiparallel beta-sheets are represented in cyan and blue. D00 shows a modified β-sandwich, opposing β-sheets are represented in green and yellow. β-strands A1 and B2 form a small separate β-sheet (dark green). The overall β-fold is disrupted in the third β-strand by an α-helix (light purple) and shortened β-strands D and E. (**c**) Topology and ribbon diagram of D0–D1. Both subdomains show a classical bacterial Ig-like domain 1 (Big1) motif, opposing antiparallel β-sheets are represented in cyan and blue.

The extended and flexible structure of the full-length intimin made structure determination with X-ray crystallography a challenge in the past. Intimin is divided into three distinct regions, the periplasmic lysin motif (LysM) domain, the membrane-embedded β-barrel and the extracellular passenger (Fig. 1a). Structural information of the three C-terminal subdomains (D1–D3), which include the Tir binding module of the extracellular passenger, have been determined^18^, as well as a crystal structure of the outer membrane-located β-barrel-domain^19^ and a solution structure of the LysM domain^22^. To establish a structural model for the membrane-embedded and extracellular part of the receptor, we determined the structures of the yet uncharacterized subdomains D00–D0 and D0–D1, which link the membrane embedded domain to the extracellular passenger domains. Each of the constructs was purified separately and characterized by X-ray crystallography. Both constructs of the extracellular subdomains, D00–D0 (PDB: 6TQD) and D0–D1 (PDB: 6TPL), crystallized in space group P1 with six (Fig. S1a) and two molecules per asymmetric unit, respectively. The data collected for the D00–D0 structure included a pseudo translation peak, which likely hampered the refinement statistics marginally, and likely the reason that the R and R_free_ values could not be optimized further for the D00–D0 dataset (Table 1).

**Table 1:** Data collection and refinement statistics.

	D00–D0	D0–D1
Data collection		
Wavelength	0.9763	0.9762
Resolution range	24.83–1.48 (1.53–1.48)	24.77–1.80 (1.86–1.80)
Space group	P1	P1
Cell dimensions		
*a, b, c* (Å)	56.0, 65.5, 79.4	33.7, 45.3, 67.6
α, β, γ (°)	77.8, 76.6, 84.8	97.1, 97.6, 99.9
Total reflections	809,651 (49,459)	125,718 (12,231)
Unique reflections	175,969 (16,162)	32,902 (3238)
Completeness (%)	98.2 (90.2)	91.4 (89.2)
I/σI	6.40 (1.5)	7.49 (2.1)
Wilson B-factor	10.7	14.9
R_merge_	0.12(0.57)	0.1014 (0.52)
R_meas_	0.15 (0.70)	0.1181 (0.60)
R_pim_	0.07 (0.38)	0.06 (0.31)
CC_1/2_	0.98 (0.35)	0.99 (0.82)
CC*	0.99 (0.72)	0.998 (0.95)
Refinement		
Reflections used in refinement	175,724 (16,141)	32,879 (3235)
Reflections used for R_free_	8656 (766)	1615 (160)
R_work_	0.17 (0.33)	0.18 (0.23)
R_free_	0.22 (0.37)	0.23 (0.30)
CC (work)	0.97 (0.34)	0.94 (0.90)
CC (free)	0.96 (0.58)	0.886 (0.85)
Number of non-hydrogen atoms	11,495	3471
Macromolecules	9329	2849
Ligands	30	7
Solvent	2136	615
RMS (bonds)	0.008	0.009
RMS (angles)	0.91	1.02
Ramachandran favored (%)	98.5	97.4
Ramachandran allowed (%)	1.5	2.6
Ramachandran outliers (%)	0.00	0.00
Rotamer outliers (%)	0.48	0.31
Clashscore	0.96	2.43
Average B-factor	18.63	26.31
Macromolecules	16.27	25.43
Ligands	29.56	30.95
Solvent	28.78	30.32
Number of TLS groups	36	13

^a^Statistics for the highest-resolution shell are shown in parentheses.

The structure of D00–D0 has an elongated conformation, spanning 78 Å between the N- and C-termini (Fig. 1b). By contrast, the crystal structure of D0–D1 exhibits a kink between the two subdomains, leading to an approximately 45° angle between the two and a distance between the N- and C-termini of only 36 Å (Fig. 1c). The D0–D1 and D0–D00 structures presented, probably only represent one of many possible conformations, as most passengers from characterized inverse autotransporters typically exhibit an extended structure^10^.

The crystal structures of each of the subdomains, D1, D0 and D00, show an expected Ig-like fold consisting of a β-sandwich of seven antiparallel β-strands. Bodelón et al.^23^, predicted for EPEC D1 a fold belonging to Ig superfamily (IgSF) type-I set with seven β-strands. Here, we show that D0 and D1 exhibit a seven-stranded strand-switched type (s-type) fold. In this fold, β-strand C’ belongs to sheet II instead of sheet I, as demonstrated in the classical c-type subtype^24^. Canonical Ig domains contain a disulfide bridge between strands B and F, which is not present in any of the intimin Big subdomains^25^. The subdomain D00 also exhibits an IgSF fold with a β-sandwich, yet it shows distinct structural differences when compared to the s-type fold seen in subdomains D0 and D1. In addition to the two common β-sheets, β-strands A1 and B2 form a separate small β-sheet due to shortened β-strands D and E. The presence of a third β-sheet in an Ig-fold is not novel as it has been previously described in Ig-domain subtype C4^25^. The β-sandwich of D00 is additionally disrupted in strand C by a short α-helix induced by residue Pro515.

Sequence analysis was performed using the profile hidden Markov model program HMMER, via the website (hmmer.org). The HMMER prediction readily detects a bacterial Ig-like domain 1 (Big1) motif in the sequence of intimin for subdomains D0 and D1, whilst no recognizable fold is predicted for the subdomain D00. Additionally, intimin D0 is structurally highly similar to D1 with a root mean square deviation (RMSD) of 0.68 Å and 0.84 Å with invasin subdomain D1 while sharing little structural conservation with the intimin subdomain D00 (RMSD: 4.68 Å). Yet, D0 and D1 share only 38% sequence identity, while D00 shares 50% sequence identity with D1 and only 25% with D0. A structural similarity search with the D00 structure on PDBeFold revealed similarity to the outermost extracellular domains of several Cadherin types (EC)^26^. Cadherin domains also consist of a β-structured IgSF fold. Similar to D00 and in contrast to D0, EC domains also exhibit a disruption of the β-sandwich by a short helix in connection with β-strand C and shortened loop between strand D and E^20^.

### The connector region between subdomain D00 and D0 shows no effect on mechanical stability

**Figure 2:**
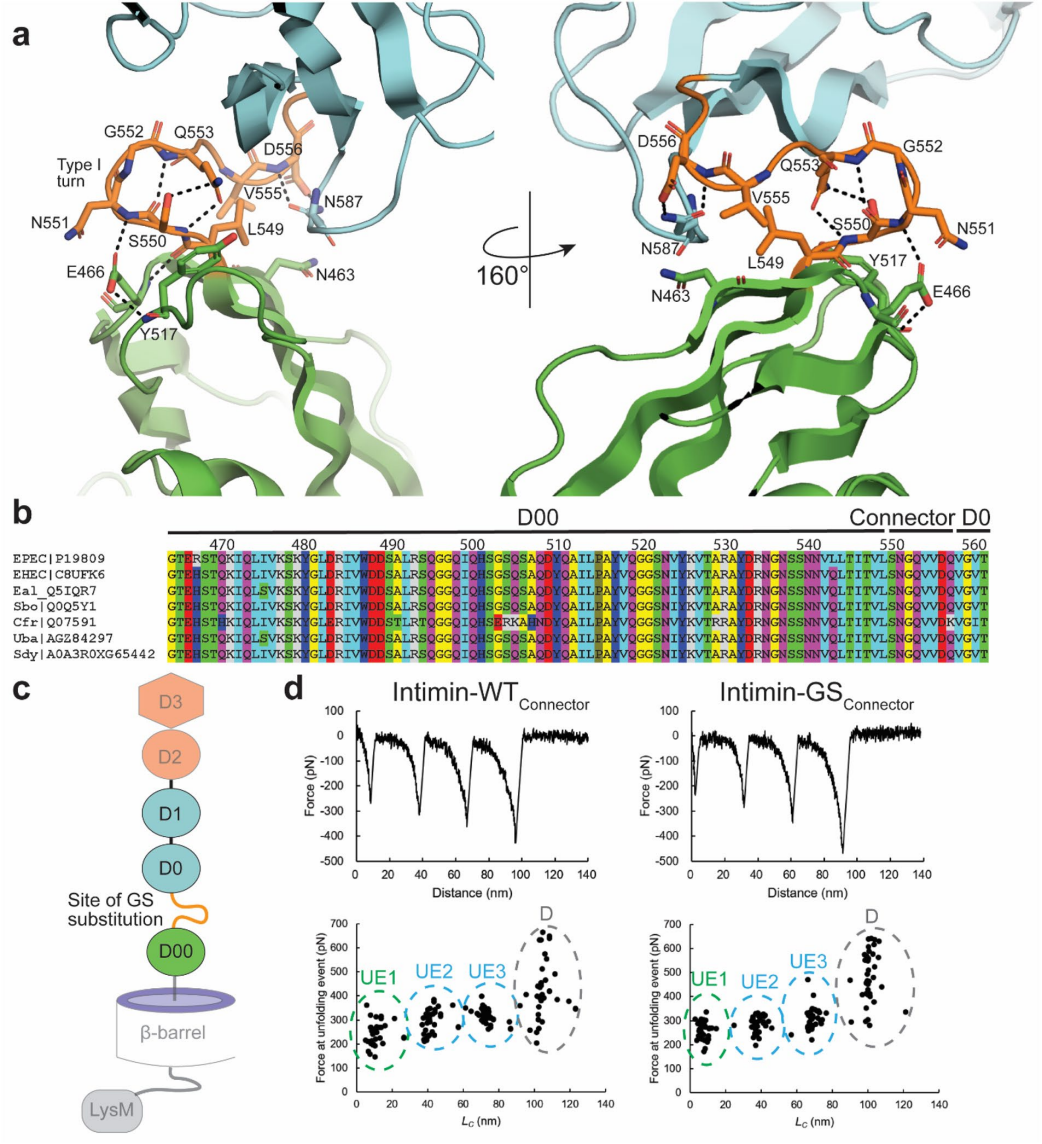
The connector region between subdomain D00 and D0 shows no effect on mechanical stability. (**a**) The connector region (orange) between D00 (green) and D0 (cyan) is represented. A network of hydrogen bonds between indicated amino acids stabilizes the extended connector, which forms a type I turn. (**b**) Alignment of the D00 subdomain, including the linker region to D0, of intimin homologues. Only intimin from EPEC (P19809) and EHEC (P11922) share 97% sequence identity (SI), EPEC intimin and the remaining sequences share less than 90% SI. Each selected sequence is shown as the bacterial species (abbreviation as followed: Eal: *Escherichia albertii*; Sbo: *Shigella boydii*; Uba: Uncultured bacterium; Sdy: *Shigella dysenteriae*; followed by the corresponding Uniprot number or NCBI accession number. The following coloring scheme is used according to the chemical functionality of the residues: Aliphatic MLIV (light blue); aromatic HFWY (dark blue), amide group NQ (purple), hydroxyl group ST (green), negatively charged DE (red), positively charged KR (light grey), small sidechain AG (yellow), P (dark green). (**c**) Scheme of intimin highlighting the connector region between D00 and D0. Connector region (orange) was replaced by GS substitution in mutational studies to analyze the role of the connector for mechanical stability. Construct used for AFM consisting of the three subdomains D00–D0–D1, as highlighted, located in *vivo* between β-barrel and passenger subdomains D2–D3 (transparent). (**d**) AFM measurements on Intimin passenger constructs D00–D0–D1. Representative force distance curves (up) and plotting of the force peaks from each unfolding event from multiple experiments against contour length (*L*_*C*_) (down) of Int-WT_connector_ (left) and Int-GS_connector_ (right) are shown. The *L*_*C*_ values were calculated for each force-distance curve by fitting to a wormlike chain model. The clusters of unfolding events (UE) are indicated and the colors match the schematic depictions in panel **b**. The unfolding events for D00, D0 and D1 assigned based on the results obtained by Leo et al*.*^16^, the unfolding events of D0 and D1 cannot be distinguished and arbitrarily assigned. Detachment of sample from the cantilever tip is indicated by (**d**).

All extracellular subdomains are connected by short sequences, which we term ‘connectors’. The connector between subdomains D00 and D0 is longer than the other connectors in the intimin passenger. The D00–D0 connector adopts an S-shaped conformation connecting the D0 and the D00 domains through a hydrogen bond network and solvent-free dry interface (Fig. 2a). Critical hydrogen bonds are formed between the amine nitrogen of Q553 and the carbonyl group of S550 backbone. The side chain of Q553 further contributes to the hydrogen bond network as its amide group forms a hydrogen bond to the hydroxy residue of the side chain of S550 and the carbonyl group of Q553 interacts with the S550 backbone. The tight interaction between these two key residues leads to the formation of a classical type I turn in the connector. The turn is further stabilized through hydrogen bonds of the neighboring amino acids, including between the carboxy group of the E466 side chain to N551 and Y517 backbone. Closer to subdomain D0, the hydrogen bond network continues between residues D556 and N587. The connector region and D00 are highly conserved within the bacteria with known intimin sequences such as *Escherichia sp.* and *Shigella boydii* (Fig. 2b). The asymmetric unit of D00–D0 crystals contained six molecules (Fig. S1a). Superimposition of all six molecules revealed small inter domain variations that indicate dynamic flexibility between the two domains (Fig. S1b, Table S4). The sidechain rotamer of all the implicated residues and thus the hydrogen bond network of the D00–D0 connector region is conserved in all six molecules (Fig. S1c, d). All the possible dimer interfaces between the individual chains where examined and revealed only water mediated contact points. A possible biological dimer interaction is not evident from the structural data; however, dimer formation cannot be ruled out.

To further analyze the stability of the connector region between D00 and D0, atomic force microscopy was performed on passenger subdomains D00–D0–D1 of wild-type intimin (Int-WT_Connector_) and a construct, in which the connector region between D00 and D0 was replaced by repetitive GS-substitutions (Int-GS_Connector_)(residues 551–557) (Fig. 2c). Both constructs showed, as expected, three unfolding events with three peaks at ~ 250 pN, ~ 290 pN, and 310 pN, representing the unfolding of three Ig-domains D00, D0 and D1 (Fig. 2d, Table S5). These values are similar to previously obtained results by Leo et al*.*^16^ and no significant differences in force peaks upon unfolding were detected between both constructs, suggesting that the connector sequence does not affect the mechanical stability of D00–D0. The unfolding force of Ig-domains was reported to be between 130–300 pN. Intimin Big-domains revealed a force between 250–300 pN and are considered to be relatively stable among Ig-domains^27^.

To test the role of the connector between D00 and D0 in a cellular environment, Int-WT_connector_ and Int-GS_Connector_ were expressed for adhesion assays. However, *E. coli* BL21 showed significantly decreased expression levels of Int-GS_Connector_ in comparison to the wild type construct (Fig. S2). Adhesion assays were not performed as no conclusive results could be obtained due to the drastically different expression levels. This was surprising as other mutations in the β-barrel and its connector region to D00 domain^13,22^ as well as deletion of a β-strand in the D00 domain typically has not lead to similarly reduced expression levels^16^. The reduced expression of Int-GS_connector_ suggests this region is important for the stability and/or secretion of the intimin passenger.

### D00–D0 shows an extended conformation in solution while D0–D1 is in equilibrium between a bent and an elongated conformation

To determine that the elongated conformation of D00–D0 was not an artefact induced by the intermolecular forces stabilized in the crystal lattice, we analyzed the structures of D00–D0 and D0–D1 in solution. Small-angle X-ray scattering (SAXS) was performed to analyze the overall shape characteristics and flexibility of D00–D0 and D0–D1.

**Figure 3:**
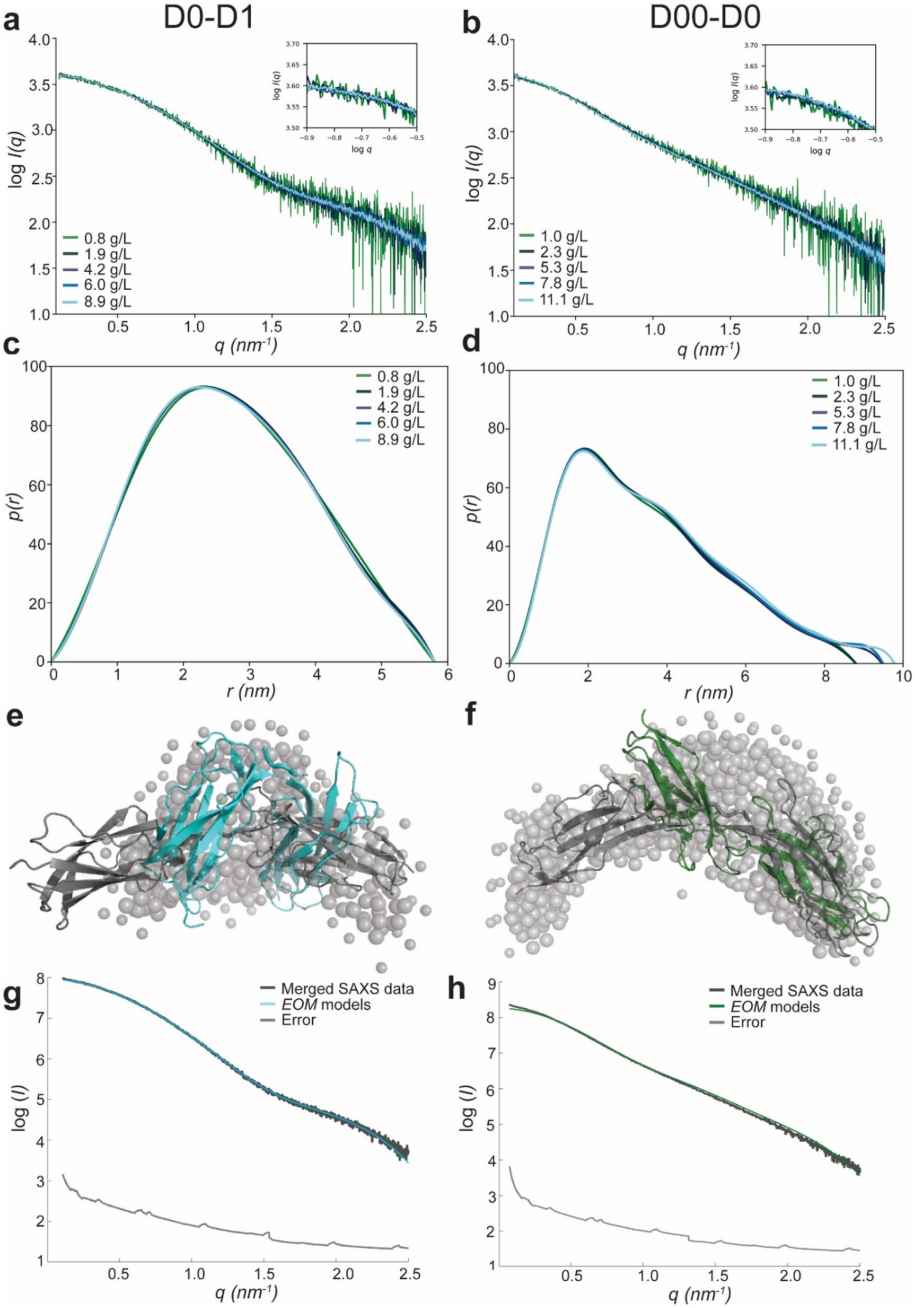
In solution D00–D0 exhibits an extended conformation while D0–D1 shows primarily a bent conformation. (**a**) SAXS scattering curves of D0–D1. (**b**) SAXS scattering curves of D00–D0. (**c**) Pair-distance distribution function *p*(*r*) of D0–D1. (**d**) Pair-distance distribution function *p*(*r*) of D00–D0. (**e**) Ab initio (grey beads) and *Ensemble Optimization Method* (*EOM*) model of bent (cyan), corresponding to the crystal structure and extended (dark grey) conformation for D0–D1. (**f**) The fitting of the *EOM* models (cyan) is shown in the graph (χ^2^ = 1.708). (**g**) Ab initio (grey beads) and *Ensemble Optimization Method* (*EOM*) model of the extended crystal structure (dark green) and *EOM* model (grey) of D00–D0. (**h**) The fitting of the *EOM* models (green) is shown in the graph (χ^2^ = 2.45).

Solution structures of D00–D0 and D0–D1 indicate no intermolecular interaction or aggregation as the SAXS curves show no changes with increasing concentration of the protein (Fig. 3a, b). The pair-distance distribution function derived for D00–D0 and D0–D1 clearly have different shapes, indicative of conformational dissimilarities (Fig. 3c, d). The maximum dimension (*D*_max_) of D0–D1 is 5.8, while D00–D0 is around 8.8–9.8 nm (Table S3), an indication that a more elongated conformation exists for D00–D0 in solution. To analyze the possible conformations in solution, both D00–D0 and D0–D1 were modeled using *Ensemble Optimization Method* (*EOM*), which is able to account for flexibility.

Ab initio modelling of D0–D1 indicated a globular and close-packed conformation (Fig. 3e) with a good data fit (χ^2^ = 2.9) (Fig. S3a). *EOM* analysis of D0–D1 revealed the presence of two conformational states, a bent and an elongated conformation. Data showed a good fit to the experimental SAXS data (χ^2^ = 1.708) (Fig. 3g; Table S6). The bent conformation fits the kinked crystal structure of D0–D1. The equilibrium is shifted towards the bent form in comparison to the extended one with a volume fraction of 0.8:0.2. Further, the presence of two different conformational states supports higher flexibility of D0–D1.

Contrary to D0–D1, ab initio modelling of D00–D0 pointed to an elongated conformation (Fig. 3f) but did not have a good fit to experimental data (χ^2^ = 18.5) (Fig. S3b). Likewise, D00–D0 *EOM* models did not have a good fit, especially at low *q-*values (χ^2^ = 11.6). Higher intensity at low *q-*values for the experimental data is an indication of the presence of larger species. *OLIGOMER* analysis of the monomer from the crystal structure and from the *EOM* analysis as well as a dimer based on the crystal structure as input was included. *OLIGOMER* results had a better fit to experimental data (χ^2^ = 2.45 for merged file) (Fig. 3h) and revealed that the majority of D00–D0 is present in the elongated conformation as shown in the crystal structure (Fig. S3c). However, the individual SAXS curves could indicate that a dimer might form at a higher volume fraction at higher concentrations (Table S7). To verify whether dimerization occurs with D00–D0 or D0–D1 in solution in the presence of increasing concentrations, SEC-MALS experiments were performed at different protein concentrations (Fig. S4a and S4b). The major peak of D00–D0 and D0–D1 for all concentrations corresponds to 22.3–23 kDa and 22.1–22.5 kDa, respectively, which fits closely to the theoretical molecular weight of a monomer of 22.8 kDa for D00–D0 and 22.6 kDa for D0–D1 (Table S8). The SEC-MALS experiments show no signs of aggregation. In comparison to SEC-MALS, SEC chromatograms of D00–D0 and D0–D1 eluted at a higher molecular weight than their theoretical one calculated from the protein sequence. Further, despite both having nearly identical theoretical molecular weights, D00–D0 eluted at a higher molecular weight than D0–D1. This is expected as the elongated shape of both molecules will increase hydrodynamic radius of the molecules, a more rigid and elongated molecule would travel slightly faster through the sephacryl matrix, than an elongated, but flexible molecule (Fig. S4d). Additionally, as SAXS experiments were performed in lower salt concentration than present during the purification process, size exclusion chromatography (SEC) was performed to analyze the effect of salt on the oligomerization or aggregation of D00–D0. SEC in different sodium chloride concentrations revealed equivalent chromatograms with a major peak corresponding to the monomer (Fig. S4c). We, therefore, conclude that D00–D0 is largely monomeric and that the apparent oligomerization of D00–D0 detected in SAXS analysis likely only exists in a negligible fraction of the sample.

### Molecular dynamics (MD) simulations support differences in flexibility between D00–D0 and D0–D1

**Figure 4:**
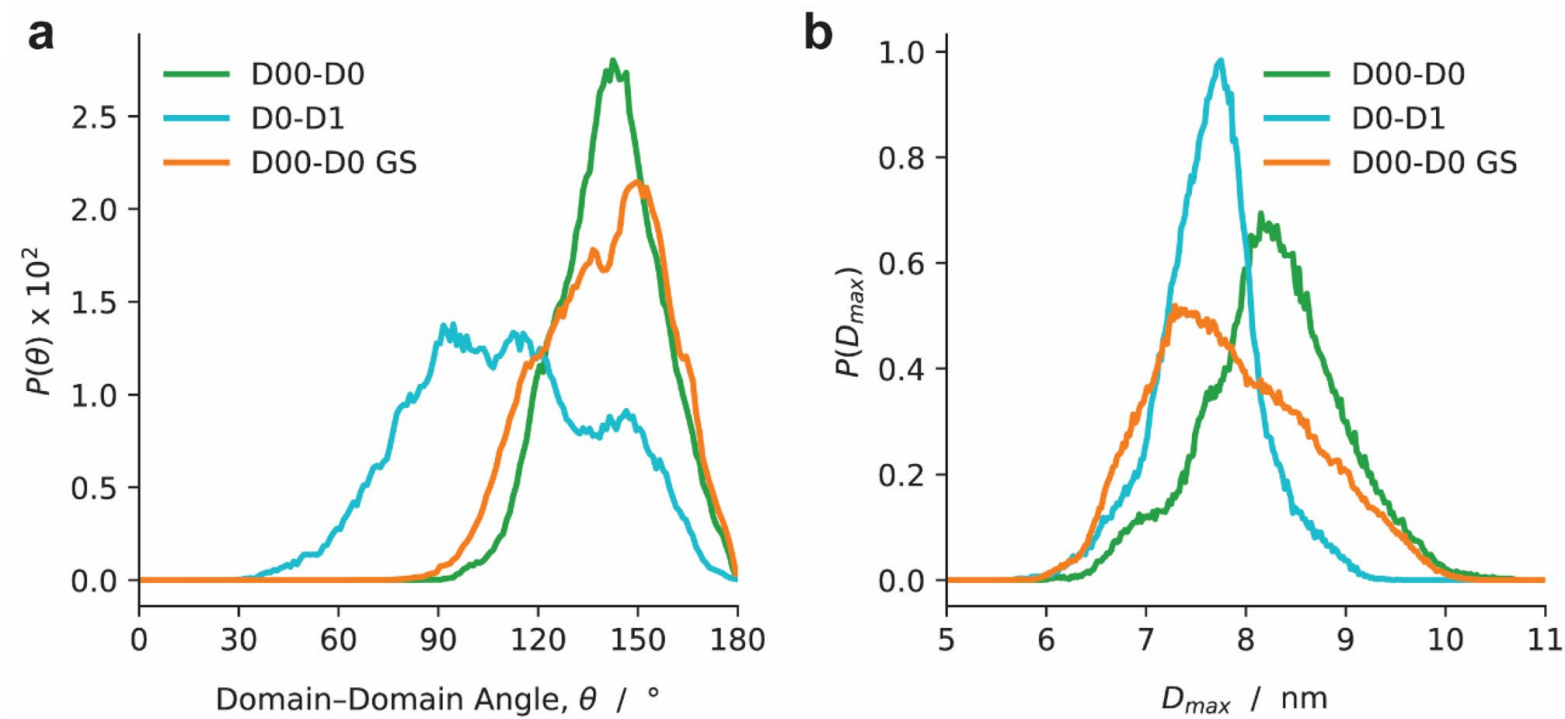
Molecular dynamics (MD) simulations support differences in flexibility between D00–D0 and D0–D1. (**a**) Probability distributions of the domain–domain angle, $\theta$ of D00–D1 (blue), D0–D1 (red) and D00–D0 GS, in which the connecter region was replaced by GS substitution (orange). (**b**) Probability distributions of maximum dimension, ${D}_{max}$, of D00–D1 (blue), D0–D1 (red) and D00–D0 GS (orange). Figure 3g, h were calculated from implicit solvent molecular dynamics simulations.

The conformational ensembles sampled by microsecond-scale MD simulations agree with SAXS findings. The conformations assumed by D00–D0 and D0–D1 are characterized in terms of the angle between the principal axes of the two domains, θ, and by the largest distance between Cα atoms, *D*_max_. The principal axis of a domain is defined as the eigenvector corresponding to the smallest eigenvalue of the inertia tensor of the backbone. Based on this definition, the crystal structures of D00–D0 and D0–D1 have θ ~ 170° and θ ~ 60°, respectively. Figure 4a shows that D00–D0 adopts extended conformations characterized by a narrow, approximately bell-shaped distribution of θ centered around 140° whereas D0–D1 is considerably more flexible and explores a wider range of domain-domain angles, including the strongly bent conformations. Accordingly, Fig. 4b indicates that the distributions of *D*_max_ estimated from MD simulations display median values that are 1 nm larger for D00–D0 than for D0–D1. Additionally, the substitution of the D00–D0 connector region with a flexible glycine-serine sequence of equal length adopts an elongated conformation with a similar bell-shaped distribution of θ as wild-type D00–D0 (Fig. 4a). However, the *D*_max_ distributions of D00–D0 GS displays a broader peak and wider range with the position of the maximum downshifted by 1 nm with respect to wild-type D00–D0 (Fig. 4b). The results indicate that, although the connector region contributes to the elongated conformation of D00–D0, it cannot be the only factor establishing the extended conformation.

The root-mean-square fluctuation (RMSF) of residues in the D00 (K450–S550), D0 (G559–V653), and D1 (T658–F751) domains of D00–D0 and D0–D1 is calculated for the backbone heavy atoms (N, Cα, C, O) after alignment of the trajectory frames to the single-domain coordinates in the corresponding crystal structures. In Figure S5, the RMSF values are superposed by shaded areas indicating the secondary structure assignment into β-strands, H-bonded turns, bends, and α-helices. Each residue is assigned to the secondary structural element identified in more than 50% of the analyzed trajectory frames according to the H-bonding and geometrical definitions of the DSSP method^28^. The most pronounced flexibility occurs in D00, particularly in regions of high curvature connecting β-strands (Fig. S5). In all domains, flexible loops are often accompanied by H-bonded turns, i.e., H-bonds between backbone carbonyl and amide groups of consecutive residues. The D00 domain shows enhanced flexibility in the loop (I475–I485) whereas the α-helix and the loop region between β-strands E and F (H501–Y510) are comparatively less mobile. D1 is the most rigid domain and, interestingly, D0 is considerably more flexible in D0–D1 than in D00–D0.

### D00 is a building block found in passengers of many inverse autotransporters

**Figure 5:**
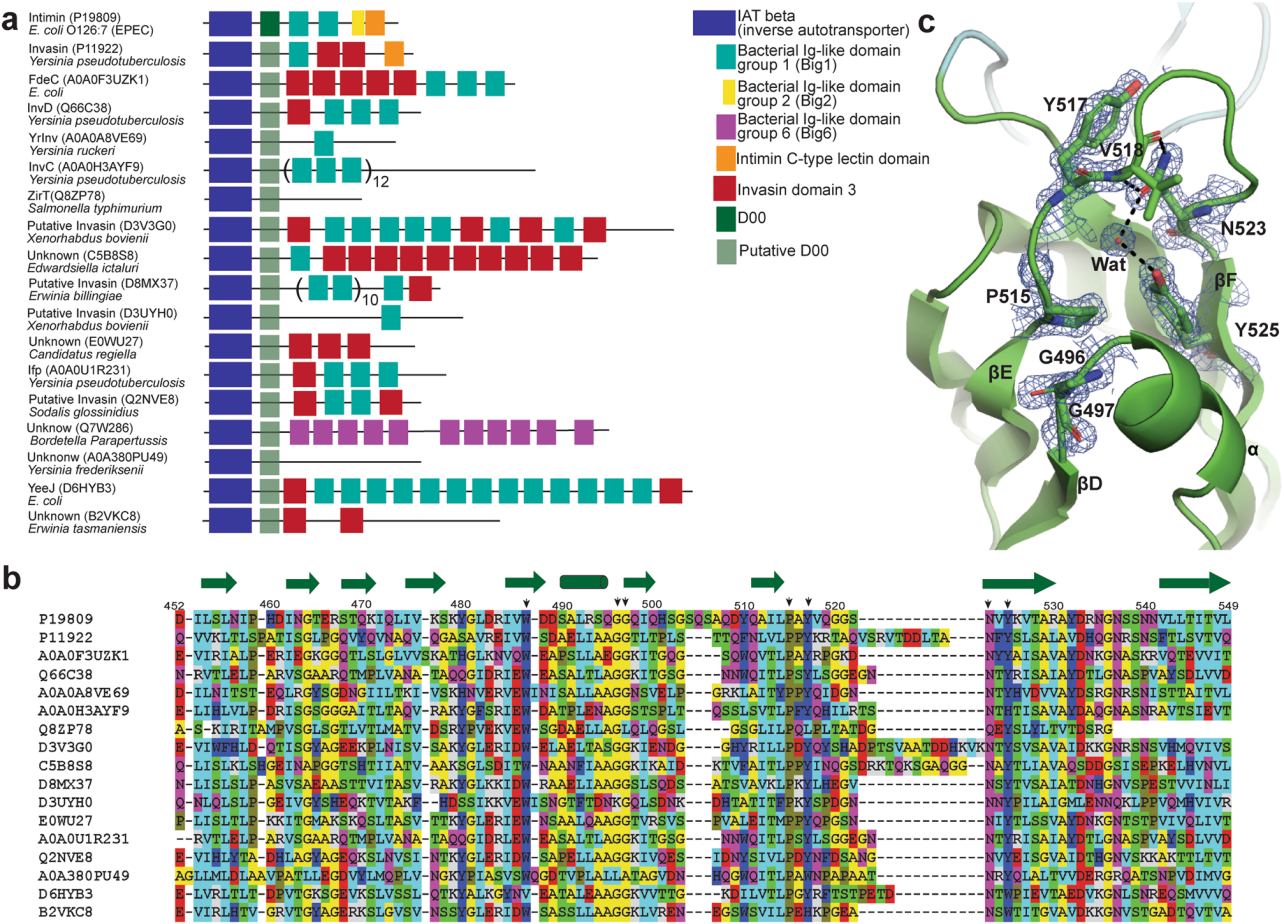
D00 is a building block found in passengers of many inverse autotransporters. (**a**) Domain prediction by HMMER of (putative) intimin, invasin and other inverse autotransporter sequences reveals repetition of the same domain types, equivalent to molecular building blocks. For most, no domain is predictable for the 100 amino acids following the β-barrel. However, we propose many inverse autotransporters contain a putative Ig-like domain homologues to the D00 domain in connection with the transmembrane β-barrel domain (translucent green). (**b**) Alignment of the D00 subdomain of intimin and invasin homologues as well as other uncharacterized inverse autotransporters. Sequences are selected with less than 70% redundancy. The colour scheme is described in Fig. 2. Green arrows represent β-strands, green cylinders α-helices as revealed by D00 crystal structure. Highly conserved amino acids, located at the top of the domain and differing from Big1-domains, are shown by a black arrow. (**c**) Amino acids in D00 are highlighted by a 2F_0_−F_c_ electron density map (blue mesh) countered at 1.5 s. The double glycine stretch allows G496 to orient the planar peptide bond with P515 in the neighbour β-strand βE. Proline residue P515 is stabilized through an interatomic aromatic-proline interaction with Y525 in β-strand F. The hydroxyl group of Y525 forms a hydrogen bond to a water molecule which by itself creates a hydrogen bond to the delta oxygen of the amido group on N523. The asparagine residue is further hydrogen bonded to the nitrogen in backbone peptide of V518 and further stabilized by a hydrogen bond between the delta nitrogen of N523 and backbone carbonyl of V518. Thereby, the amido group of N523 is forced into a planar conformation with the conjugated side chain of Y517 kept within a 3.5 Å distance. A similar structural interaction is likely present in the listed sequences in (**b**).

In recent years, the range of the inverse autotransporter family in bacteria has been shown to be significantly larger than previously recognized and also shows a wider variety of protein domains and architectures than that of just close intimin and invasin homologues^29^. Most extracellular domains of inverse autotransporters contain certain subdomain types in different amounts and orders, reminiscent of different combinations of the same building blocks (Fig. 5a). This is similar to what has been observed for type Vc secretion system or trimeric autotransporter adhesins^30^. Interestingly, for many of the (predicted) inverse autotransporters, only a poor structural domain prediction is available for the sequence (ca. 100 residues) directly after the membrane-embedded β-barrel (Fig. 5a)^31^. This has led, until recently, to the conclusion that no folded domain might be present^19^. However, sensitive homology-based searches using HHPred^32^ suggested that multiple inverse autotransporters contain a D00-like IgSF domain at the N-terminus of the passenger^16^. Due to the similarity to the domain prediction of intimin subdomain D00, we performed an alignment on this region and identified conservation of several residues separated from the overall Ig-fold (Fig. 5b). Two consecutive glycine residues (G496, G497) are conserved in close structural proximity to the proline residue (P515), followed by a tyrosine (Y517) or tryptophan. An asparagine (N523) and a second tyrosine (Y525)/tryptophan are further conserved. Additionally, D00 contains a single tryptophan residue (W487), which is present among nearly all selected inverse autotransporter sequences. The described residues are not conserved among all inverse autotransporters, but most of the representatives contain them. In intimin D00, these conserved residues are located in the loop region of β-strand E and F near the short helix element (Fig. 5c). The double glycine stretch allows G496 to orient the planar peptide bond with P515 in the neighboring β-strand E. Proline residue P515 is stabilized through an interatomic aromatic-proline interaction with Y525 in β-strand F^33^. The hydroxy group of Y525 forms a hydrogen bond to a water molecule, which by itself creates a hydrogen bond to the δ-oxygen of the amido group on N523. The asparagine residue is hydrogen-bonded to the nitrogen in backbone peptide of V518 and further stabilized by a hydrogen bond between the δ-nitrogen of N523 and backbone carbonyl of V518. Thereby, the amido group of N523 is forced into a planar conformation with the conjugated side chain of Y517 kept within a 3.5 Å distance (Fig. 5c).

### Rigidification of D00–D0 increases the radius of reach of intimin

**Figure 6:**
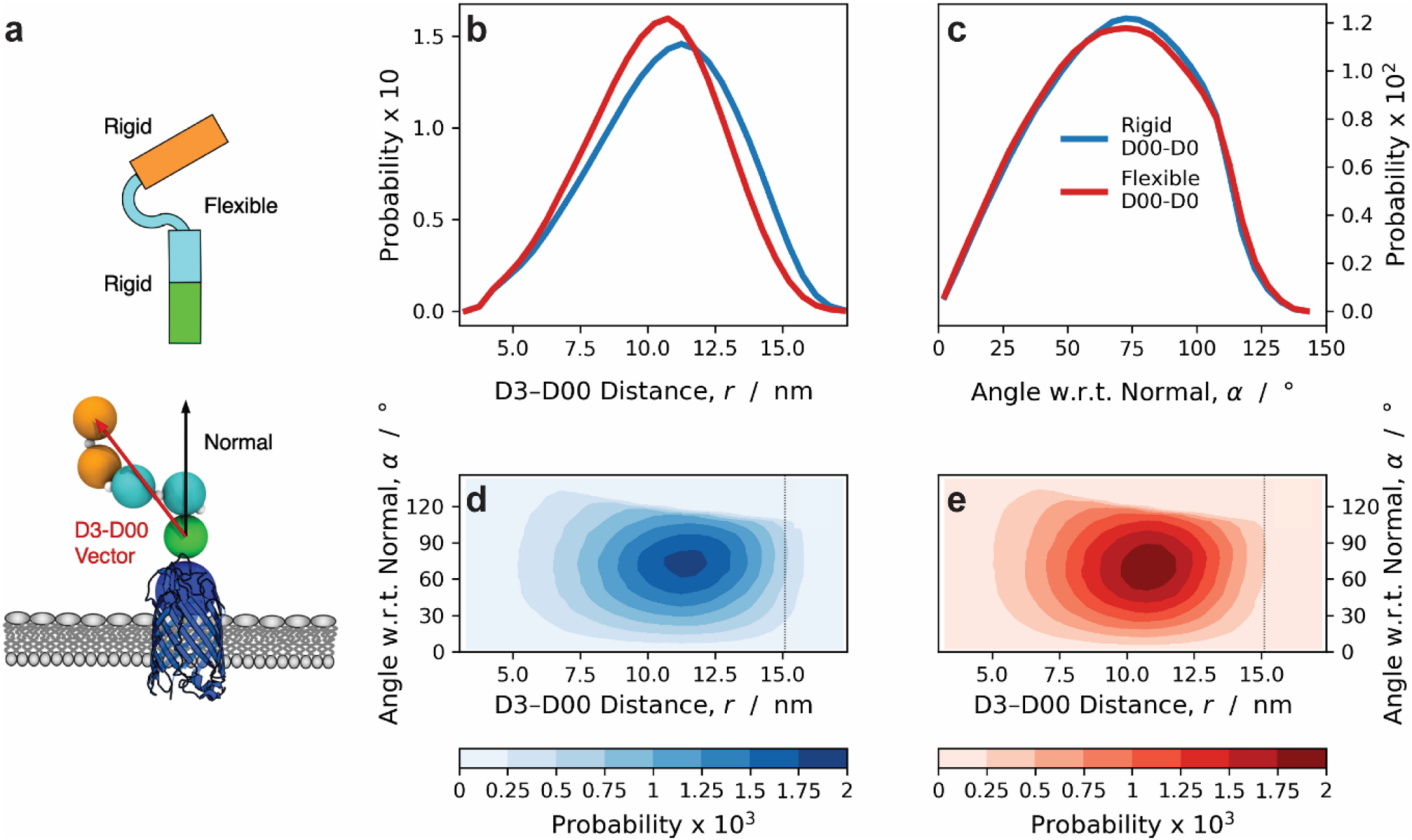
MC simulations indicate that the rigidity of D00–D0 increases the radius of reach of intimin. (**a**) Cartoon representation (upper) and visualization of the coarse-grained model (lower) of the extracellular passenger domain of intimin. In the cartoon, the rigid D00–D1 and D2–D3 domains are depicted as rectangles, whereas the flexible D0–D1 and D1–D2 connectors are represented by curves. The β-barrel, D00, D0–D1 and D2–D3 domains are shown in blue, green, cyan and orange, respectively. The white beads of the coarse-grained model represent the linker regions. The red and black arrows indicate the D3–D00 vector and the normal to the membrane surface, respectively. Probability distributions of (**b**) the distance between D3 and D00 and (**c**) of the angle between the D00–D3 vector and the normal to the membrane surface obtained from coarse-grained simulations modelling the D00–C1–D0 angle as a flexible (red lines) or rigid (blue lines) harmonic potential. 2D probability distributions as a function of the radius of reach and orientation of the D00–D3 vector obtained using a rigid (**d**) or a flexible (**e**) D00–C1–D0 angle.

To investigate the influence of the conformational rigidity of D00–D0, we developed a coarse-grained model of the extracellular passenger of intimin. The elongated protein is modelled by spherical beads located at the centers of mass of the folded domains and of the connector regions (Fig. 6a). The D00–C1, D2–C4 and D3–C4 bonds are modelled based on the all-atom data for D00 whereas the remaining bonds reproduce the all-atom distribution for the distance between D0 and the connector region of D0–D1 (Fig. S6b). Triplets of beads interact either via rigid or flexible harmonic angular potentials, which are parametrized against the all-atom distributions of the domain-domain angles of D00–D0 and D0–D1, respectively (Fig. S6a). D0–C2–D1 and D1–C3–D2 are flexible angles, whereas C1–D0–C2, C2–D1–C3, C3–D2–C4 and D2–C4–D3 are rigid.

We performed Metropolis Monte Carlo simulations of the coarse-grained intimin, modelling D00–C1–D0 either as a rigid or as a flexible angle. Figure 6b, c show probability distributions of the distance between D3 and D00, $r$, separation and of the angle between the D00–D3 vector and the normal to the membrane surface, α, for the rigid (blue lines) and the flexible (red lines) D00–D0 angles. Figure 6d, e show the corresponding 2D probability distribution as a function of both the radius of reach, *r*, and the of the multi-domain protein, α.

These results indicate that the stiffness of D00–D0 negligibly affects the orientation of the extracellular passenger with respect to the surface, whereas it significantly increases the radius of reach of the receptor.

## Discussion

**Figure 7:**
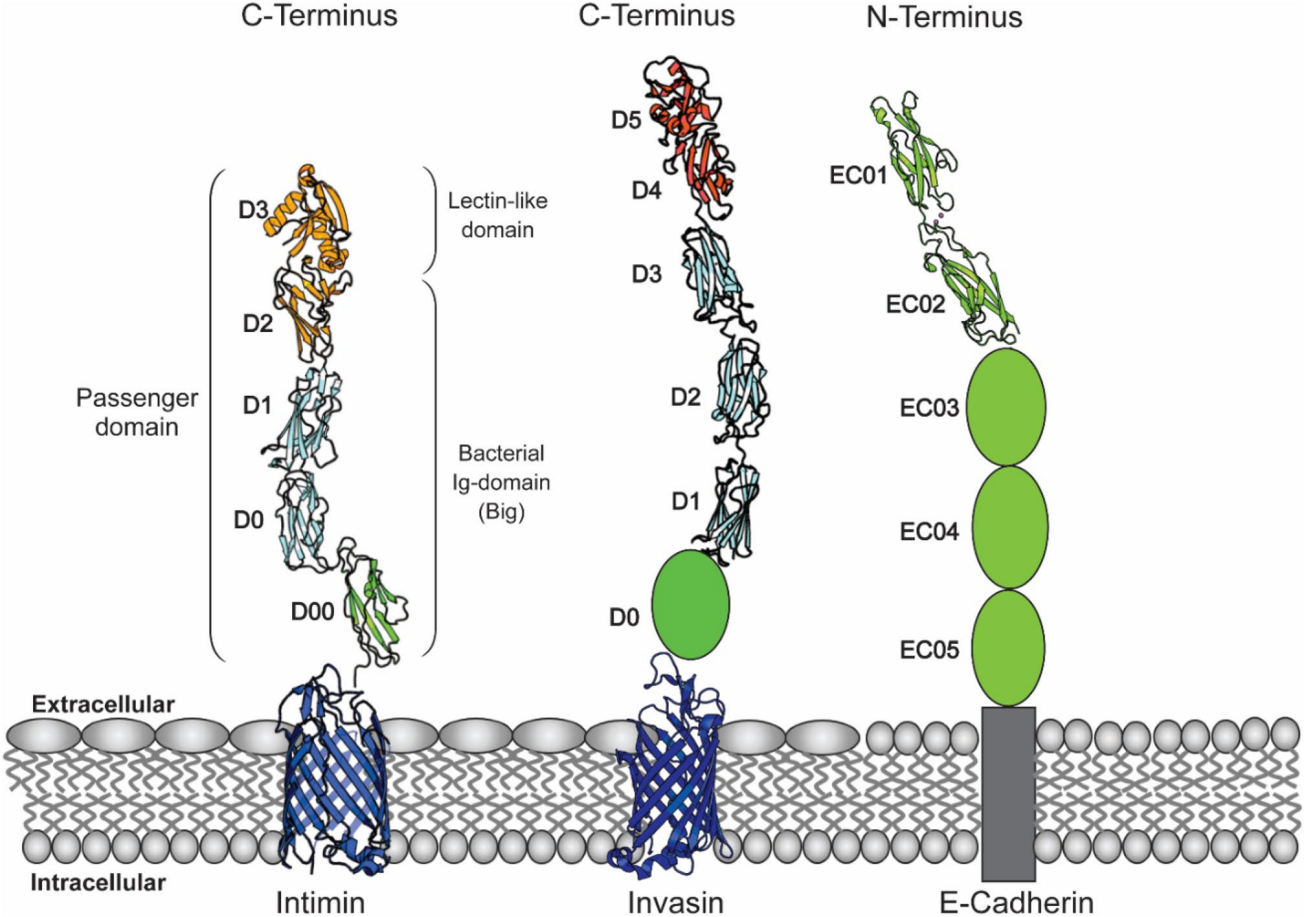
Intimin and invasin passengers show an equivalent structural composition and similarity to E-cadherin extracellular extension. Structural model of intimin exhibiting the β-barrel domain located in the outer membrane (PDB: 4E1S; blue) connected to the extracellular passenger domain consisting of four Big subdomains: D00–D0 (PDB: 6TQD; D0: cyan; D00: green) and D1–D2 connected to lectin-like domain D3 (PDB: 1F02; D1: cyan; D2–D3: yellow). Invasin exhibits high structural similarity to intimin, consisting of membrane-located β-barrel (PDB: 4E1T, blue) and a long stalk of Ig-domains with a lectin-like receptor superdomain at the C-terminus (PDB:1CWV, D1–D3: cyan, D4–D5: red). Based on our alignment, we propose an additional domain (D0) at the N-terminus of the invasin passenger, equivalent to intimin subdomain D00. The structure of this domain is unsolved, but our work suggests a similar structure to intimin D00. Further, D00 revealed structural similarity to the extracellular domains of cadherins (EC) which are represented here by E-cadherin (PDB: 2QVF). In comparison to intimin and invasin, there is no structural difference in the domain composition along the extracellular stalk of cadherins. The rigidity and flexibility of Cadherins are regulated by calcium binding in the connector regions of EC domains.

The tight attachment to the host cell under fluid flow conditions is a major obstacle for bacterial cells during adhesion. Most bacteria therefore have adhesion molecules able to withstand the shear forces present in a fluid environment and maintain a strong attachment^34^. Intimin, an adhesion protein essential for EPEC and EHEC infection in the intestinal environment, is required to also withstand these physical conditions, especially in its extracellular passenger^35^. In this study, we investigated the structure and organization of the subdomains of the passenger region regarding physical properties that could play a role in promoting attachment during the adhesion process. We propose a structural model of the entire passenger of *E. coli* intimin and its connection to the bacterial outer membrane, sharing a common structural organization with invasin (Fig. 7). Structures of subdomains D00–D0 and D0–D1 obtained in this study were modelled in connection to isolated structures of the C-terminal passenger domain (PDB: 1F02) and the translocation unit (PDB: 4E1S) to obtain a model for the complete passenger. We hypothesize that the repeated Big domains of the extracellular passenger are divided into different subregions depending on the connectors between the individual Big domains. This domain organization would allow the passenger region to varying in rigidity and flexibility. Although the functional role of these consecutive variations in flexibility is unclear, we hypothesize that they promote increased binding probability of the passenger and intimate attachment (Fig. 6b–e).

We propose that a D00-like Ig domain is a common element present in most inverse autotransporters, including the passenger of invasin. The invasin D0 domain would thus be equivalent to intimin D00. The list includes, next to intimin and invasin homologues, other inverse autotransporters such as FdeC from *E. coli*^36^ or inverse autotransporters YrInv and YrIlm from the fish pathogen *Yersinia ruckeri*^37,38^. Our study highlights that D00 should be included as a building block in domain prediction tools. Previous results demonstrated that the D00 domain is not crucial for the intimin autotransport process and an intimin mutant in which the D00 domain was deleted showed similar adhesion to HeLa cells compared to the wild-type protein^16^. However, it is positioning directly after the translocator at the membrane interface supports our hypothesis that D00 acts as a rigid stabilizing factor for the long rod-shaped extracellular domain. The present study strengthens this view and shows the importance of the D00–D0 connector, as changing this region to a flexible GS linker resulted in a dramatic reduction in protein levels. Despite an overall Ig-fold, D00 exhibited differences to the other Big-domains in the passenger region, while we detected structural similarity to cadherin EC domains (Fig. 7). We propose to place the D00-like Ig domains in a unique bacterial Ig domain group, distinct from the bacterial Ig-like group 1, that D1 and D0 belong to.

The following subdomains, D0–D1, extend the stalk of the passenger. In other inverse autotransporters, the number of Big domains in this region can vary extensively, ranging from two to 47^39^. The reasons for these variations are still unknown, but it was hypothesized that it is important for spanning the bacterial surface structures, so that the functional domain (adhesin domain) can be displayed for interaction with the host^39^. The C-terminus of the extracellular passenger region consists of the actual receptor module, the superdomain D2–D3 in intimin, which directly interacts with the Tir receptor on the host cell surface. Regarding the overall structure of the extracellular passenger, our results show that the extended string of tandem Ig-domains exhibits an internal twist with each domain turned approximately 180° degrees in comparison to the previous one, making the entire passenger resemble a helix. Similar formation of a higher-order structure of a superhelical rod-like macrostructure consisting of hundreds of Ig-like subdomains is present in Type 1 pili, important for bacterial cell adhesion^23^. In the case of type 1 pilus, the superhelical structure can unwind, thus reducing shear stress upon receptor binding under fluid forces^40^.

**Figure 8:**
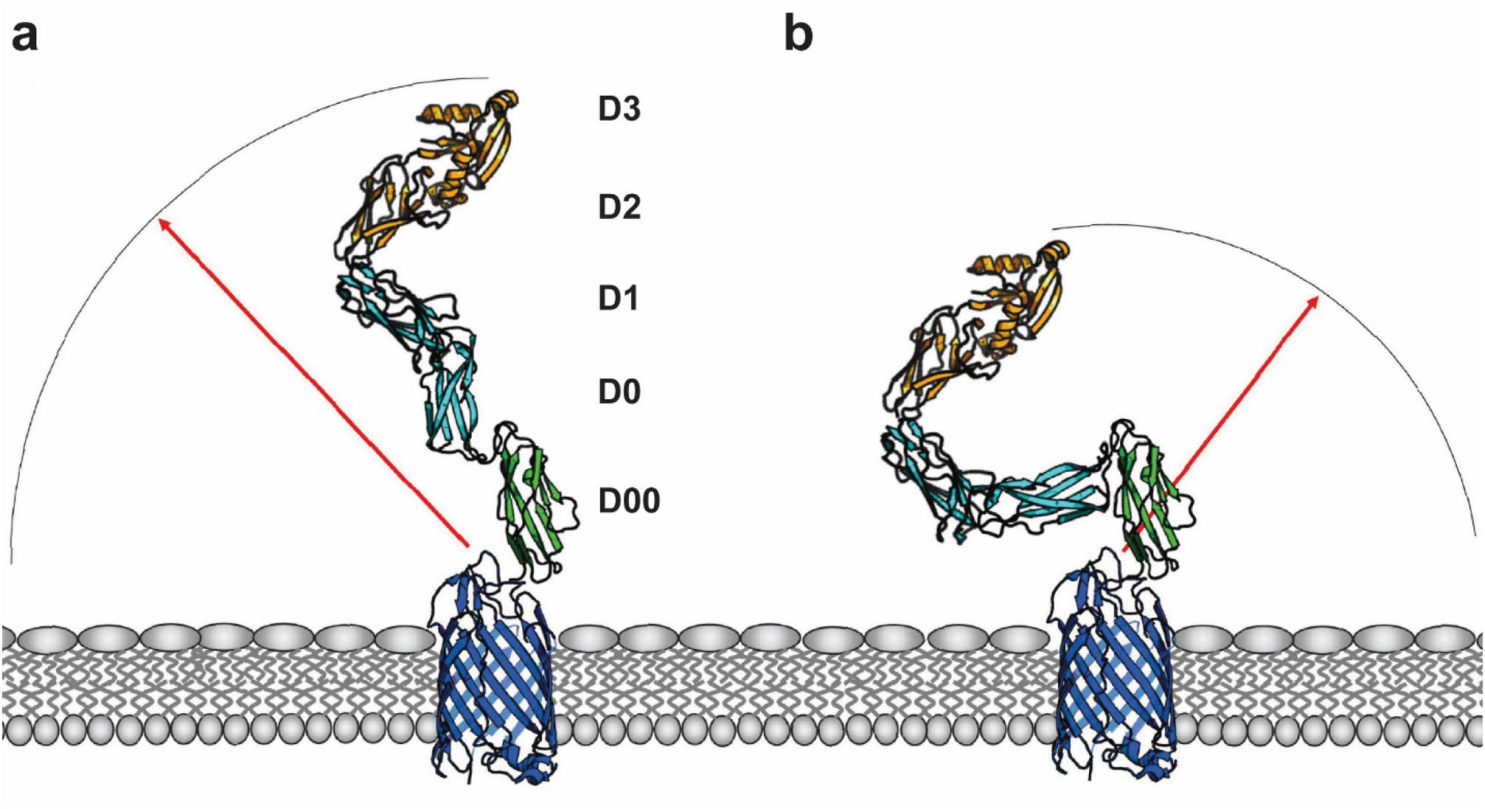
Model for how regions of rigidity and flexibility in the intimin passenger promote adhesion and intimate attachment. (**a**) The D00–D0 domains form a rigid unit, which projects the intimin passenger further from the bacterial surface, thus increasing the average radius of its reach (red arrow) and sphere of action (arc). (**b**) In the hypothetical situation where the connector between D00–D0 allows similar flexibility as seen experimentally for D0–D1, the average radius of reach and sphere of action are reduced.

Our work indicated differences in rigidity and flexibility between the different extracellular domains. D00–D0 revealed an elongated and more rigid conformation while D0–D1 displayed increased flexibility with higher probability to be present in a bent conformation in solution, equivalent to the obtained crystal structures. This rigidity is reminiscent of the inflexibility seen between D3 and D2 in the solution structure of the C-terminus of intimin^41^. By contrast, the D1–D2 connector is flexible in the solution structure^41^, similar to what we observed for D0–D1. Therefore, we propose a model of the intimin passenger in which D00–D0 and D2–D3 form functional units of high rigidity while the stalk subdomains D0–D1–D2 show higher flexibility which could be beneficial during the binding process (Figs. 6, 8). MC simulations supported the notion that a rigid base in the intimin passenger, represented by D00–D0, increases the radius of reach and shifts the orientation of intimin in relation to the mammalian host cell. This could accelerate the binding process of intimin and the Tir receptor, presented on the mammalian host cell, and potentially increase the efficiency of a successful adhesion event (Fig. 8). The flexibility demonstrated by the D0–D1 and D1–D2 connectors is also likely to be functionally important. The crystal structure of the intimin-Tir complex shows a large angle, close to 180°, between the two proteins. For the rigid D2–D3 domain to approach at this angle, the intimin passenger must be able to bend. This configuration also brings the host cell membrane and the bacterial outer membrane into close proximity. It promotes the two passengers of one intimin monomer to interact with separate Tir molecules, rather than a single dimer. This is necessary for receptor clustering and downstream signaling events leading to the formation of the actin pedestal^22,42^.

Interestingly, also members of the cadherin family, a family of adhesion proteins containing long sequences of IgSF domains in their extracellular domains, contain both extended and bent elements^43^, similar to the structural conformation obtained for D00–D0 and D0–D1, respectively. In their functional forms, the cadherin domains are rigidified by Ca^2+^-binding sites in the domain linker regions. Yet, structural forms of the T-cadherin domains without Ca^2+^-bound showed a strong bend between neighboring domains. The presence of Ca^2+^-binding sites allows regulation of the flexibility of the extracellular adhesion domains. For some members, like Protocadherin-15, the overall rigid conformation is locally permanently disturbed through a calcium-free linker between the two neighboring domains, in which the Ca^2+^ binding sites were mutated. It was proposed that this structural feature confers soft elasticity in an otherwise rigid tip link, allowing a smoother response to low forces applied as the molecule will rather extend than unfold. Another example in which the presence of elastic elements disrupted a linear rod conformation was found in titin, a muscle protein submitted to shear stress and involved in mediating elasticity^44^. In this case, elasticity was increased through bending and twisting of neighboring IgSF domains.

In summary, our findings on structural and biophysical similarities and differences of subdomains on the extracellular passenger region of intimin can contribute to our general understanding on functional regions along the often neglected adhesin stalk and how receptor binding can be affected through variations in rigidity and flexibility.

## Material and methods

Chemicals were obtained as grade BioUltra from Sigma-Aldrich unless otherwise stated.

### Constructs of D00–D0 and D00–D1

The D00–D0 domains of intimin were amplified using Q5 polymerase (New England Biolabs) and pIBA2-Int-Strep as template^16^. Primers were designed to introduce an N-terminal hexahistidine tag. D00–D0 were then cloned into the expression vector pAKS-IBA3 (IBA GmbH) using Gibson assembly^45^. The reaction mix was transformed into chemically competent TOP10 cells (ThermoFisher), and transformants were selected on lysogeny broth (LB) medium supplemented with ampicillin (100 µg/ml)^46^. Positive colonies were screened for using colony PCR, and a PCR-positive clone was verified by Sanger sequencing (Eurofins).

To introduce a tobacco etch virus (TEV) protease cleavage site after the His-tag, site-directed mutagenesis was employed^47^. Once the insertion had been verified by sequencing, the plasmid was amplified from the TEV site and from after the D00–D0 insert to produce a linearized plasmid with a His-tag followed by a TEV site on one end and the stop codon on the other. Into this, we inserted the sequence coding for the D0–D1 domains, amplified as above. The cloning was performed as outlined above. These procedures resulted in two plasmids, pIBA3-His-TEV-IntD00–D0 and pIBA3-His-TEV-IntD0–D1.

To increase flexibility in the connector, the connector sequence (residues 551–557) was replaced by a glycine-serine sequence of equal length (GSGSGSG). The exchange was done by site-directed mutagenesis^47^. This was done for both the three-domain construct pIBA3-IntD00–D1Cys^16^ to produce pIBA3-IntD00–D1Cys-GS_connector_ and in full-length intimin (pIBA2-Int-Strep) to produce pIBA-Int-GS_connector_-Strep. The mutations were verified by sequencing.

All primer sequences are given in Table S1.

### Expression and purification

The expression and purification of all constructs were performed according to the protocol described in^16^. D00–D0 or D0–D1 inserted into pIBA3-vectors, were transformed into *E. coli* BL21Gold(DE3) (Novagen) and plated on LB medium with 1.5% agar and 100 μg/ml ampicillin. Colonies of transformed BL21Gold(DE3) cells were inoculated in LB medium containing 100 μg/ml ampicillin and incubated at 37 °C for 16 h (h). The following day, 1% overnight culture was added to ZYP medium (10 g tryptone, 5 g yeast extract, 5 g sodium chloride in 1 L ddH_2_O) enriched with 100 μg/ml ampicillin and incubated with shaking at 37 °C until the optical density at 600 nm (OD_600nm_) reached 0.5–0.7. Protein expression was induced with anhydrotetracycline (ATCH) (IBA GmbH) at a final concentration of 0.2 ug/ml. The cells were further grown at 18 °C for 16 h. Cells were harvested by centrifugation at 7000×*g* for 10 min. Cell pellets were stored at − 20 °C until use. Thawed cell pellets were resuspended at 1:10 ratio in buffer A (10 mM HEPES–NaOH pH 7.5, 400 mM NaCl, 1 mM Phenylmethylsulfonyl fluoride (PMSF), 1 μg/ml DNase) and lysed using a high-pressure homogenizer (C5 model, Avestin, Germany) (3 passes at 15,000 psi). Cellular debris and the membrane fraction were removed by centrifugation at 100,000 * g for 45 min. The clarified supernatant was applied to a 5 ml HisTrap HP column (GE Healthcare). The column was washed with 10 column volumes (CV) in buffer A supplemented with 30 mM imidazole. The protein was eluted with buffer A supplemented with 120 mM imidazole and the fractions containing the D00–D0 or D0–D1 protein were pooled separately. His-tagged TEV protease purified in-house^48^ was added to the protein solution in a 1:28.5 (w/w) ratio and dialyzed against 10 mM HEPES–NaOH pH 7.5, 400 mM NaCl, 5 mM β-mercaptoethanol (βME) for 16 h at 4 °C. The dialyzed sample was passed through a Histrap HP column equilibrated in buffer A. The flow-through was collected and concentrated (Centrifugal concentrator, 10 kDa molecular weight cut off (VivaSpin)). The purity of the sample was assessed by SDS-PAGE and size exclusion chromatography, the Superdex 75 10/300 GL column (GE, cat. # 29148721) was equilibrated with 10 mM HEPES-NaOH pH 7.5, 400 mM NaCl.

For atomic force microscopy experiments, proteins D00–D1 and D00–D1-GS_connector_ with C-terminal cysteines were produced and purified as described in^16^.

### Crystallization

D00–D0 or D0–D1 constructs were further concentrated to 50 mg/ml and 80 mg/ml, respectively, to reach supersaturation and induce crystal formation. Crystallization experiments were performed with the Index screen (Hampton Research), and inhouse screens. Drops were set up with 1 μl of protein and 1 μl precipitant solution in 24-well plate as hanging drops. The wells were sealed with immersion oil (Sigma cat. # 56822). Plates were incubated at 18 °C. Initial crystals of D00–D0 appeared in three days. The best D00–D0 crystals were grown using a reservoir solution of 0.15 M potassium bromide and 30% (w/v) polyethylene glycol monomethyl ether 2000 (PEG2000mme). The best crystals of D0–D1 were obtained in 0.1 M tris, pH 8.5; 0.2 M magnesium chloride hexahydrate and 25% polyethylene glycol 3350 (PEG3350). Crystallization had to be induced by the addition of 4% glycerol in the reservoir to promote vapor diffusion; crystals appeared after one day. Crystals were harvested using mounted CryoLoops (Hampton Research) and flash frozen in liquid nitrogen. Data sets were collected at DESY Hamburg, Germany, using the PETRA III beamline P14 (see Table 1).

### Data processing

Molecular replacement using the program Phaser^49^ was used to solve the structures of D00–D0 and D0–D1 with D1 domain (PDB: 1F02)^18^ as an initial search model. The D0 domain, from the refined structure of D0–D1 was used as search model in the D00–D0 dataset. The data for the D00–D0 showed a presence of a pseudo translation peak at position (0,0,0.3) given in the output from Xtriage^50^, it was necessary to include the pseudo translational peak vector in phaser to obtain a partial solution that correctly placed the six D0 domain and allowed the D00–D0 structure to be automatically build using the AutoBuild wizard within the PHENIX package^50^. The structures were refined using the phenix-refine^50^ and REFMAC^51^, final model building was performed in Coot^52^. Data collection and refinement statistics are summarized in Table 1. Molecular graphics were presented with The PyMOL Molecular Graphics System, Version 2.2r7pre, Schrödinger, LLC.

### Small angle X-ray scattering (SAXS)

Purified D00–D0 and D0–D1 were dialyzed against 10 mM HEPES-NaOH, pH 7.5 and 100 mM KCl and concentrated to ca. 28 mg/ml in a centrifugal concentrator (10 kDa molecular weight cut off (VivaSpin)). The buffer that passed through the filter was collected and used as a blank for the SAXS measurement. SAXS data were collected at the P12 beamline at the Petra III storage ring (DESY, Hamburg DE)^53^ (for experimental details see Table S2). Structural parameters, including the radius of gyration (*R*_g_) and maximum dimension (*D*_max_), were derived from the experimental data with the graphical data analysis program *PRIMUSQT*^54^*. *Ab initio models for both proteins were obtained from *GASBOR*^55^*.* The conformational polydispersity of each protein was studied using *Ensemble Optimization Method* (*EOM*)^56,57^, which consists of two programs *RANCH* and *GAJOE. RANCH* was employed to create 10,000 random conformations (genes) by using two domains (D452–L549 and G559–Q656) from the crystal structure. *GAJOE* was used to select ensembles of conformations that have a better fit to the experimental data and was run ten times for each protein. The fit was done against merged SAXS curves. For the merged curve, data for the lower *q*-values were taken from the low protein concentrations SAXS curves, while data for the higher *q*-values were taken from, the higher protein concentrations. This allowed to in order to minimize noise at high *q*-values and remove the contribution of interparticle interaction at low *q*. For D00–D0 SAXS data, *OLIGOMER* was performed, using in total 13 components: all the models from *GAJOE*, the crystal structure, and two dimers (chains A, B and A, F) extracted from the crystal structures (Fig. S1a). For OLIGOMER we initially used both AB and AF dimers. However, the AB dimer was rejected as the volume fraction approached zero for this structure, therefore in the final run only the AF dimer was used. *FFMAKER*^54^ was used to create an input file for *OLIGOMER* with a form factor for each component. Experimental details are shown in Table S2 and S3. Measurements were taken from distinct samples.

### Molecular simulations

Molecular dynamics (MD) simulations are performed using the AMBER ff14SB force field^58^ and the GB7 implicit solvent model^59^. The ionic strength of the solution is set to 0.15 M and modelled within the Debye–Hückel approximation^60^. After energy minimization of the respective crystal structures, D00–D0 and D0–D1 are simulated for 3.0 µs using the AMBER16 MD package in the *NVT* ensemble at 298 K maintained by Langevin dynamics with a collision frequency of 20 ps^−1^. The integration time step is 2 fs, and all bonds involving hydrogen atoms are constrained using the SHAKE algorithm^61^. Secondary structure assignments are performed using the DSSP algorithm^28^ implemented in MDTraj^62^. The first 200 ns of the trajectories are excluded from the analyses based on the time evolution of the RMSD with respect to the crystal structure (Fig. 6).

Metropolis Monte Carlo (MC) simulations are performed using the Faunus framework^63^. The coarse-grained model of the extracellular passenger of intimin consists of five beads of diameter 4 nm separated by four smaller connector beads of diameter 1 nm (Fig. 6a). The beads are connected by harmonic bonds parametrized by fitting a Gaussian function to the distributions of the mass-centre distances between the domains and the connector in the all-atom trajectory of D00–D0 (Fig. S7b). The D00–D0 bond has a force constant ${k}_{b},$ of 1 kJ/mol and equilibrium distance $r_{eq}$, of 2.6 nm whereas all the other bonds have ${k}_{b}=0.5$ kJ/mol an, ${r}_{eq}=2.1$ nm. Angular interactions are modelled by harmonic potentials derived by fitting a Gaussian function to the probability distributions of the domain-domain angles of D00–D0 (rigid) and D0–D1 (flexible) (Fig. S7a). Rigid angles have force constants, ${k}_{\theta }$, of 30 kJ/mol and equilibrium angle, ${\theta }_{eq}$, of $150^\circ$ while flexible angles have ${k}_{\theta }=2$ kJ/mol and ${\theta }_{eq}=110^\circ$. The mass-centre of the D00 bead is fixed at 5 nm above a hard wall, which represents the membrane surface located in the *XY*-plane. To account for the excluded volume of the $\beta$-barrel domain, an extra bead of diameter 5 nm is positioned between the membrane and the D00 bead, at a distance of 0.5 nm from the hard wall. The other beads translate in the volume above the surface, sampling the conformational space of the coarse-grained adhesin model at 298 K. Nonbonded bead-bead interactions are modeled via the Weeks-Chandler-Andersen potential^64^. The probability density distributions of the D3–D00 distance, $P\left(r\right)$, and of the angle formed by the D3–D00 vector with the membrane normal, $P\left(\alpha \right),$ as well as the 2D probability distribution $P\left(r,\alpha \right)$, distribution $P\left(r,\alpha \right)$, are normalized so that ${\int }_{0}^{\infty }{\mathrm{d}}r P\left(r\right)=1$, ${\int }_{{0}^{\circ }}^{{180}^{\circ }}{\mathrm{d}}\alpha P\left(\alpha \right) =1$ and ${\int }_{0}^{\infty }{\int }_{{0}^{\circ }}^{{180}^{\circ }}{\mathrm{d}}r {\mathrm{d}}\alpha P\left(r,\alpha \right) =1$. A Jupyter notebook in ipynb (Supplementary Data 1) and HTML formats (Supplementary Data 2) detailing simulations and analyses of the coarse-grained model are provided as supplementary data. The simulation data, scripts and input files are available on GitHub at https://github.com/gitesei/SI-intimin.

### Atomic force microscopy

Atomic force microscopy (AFM) was carried out as previously described^16^ with slight modifications. To prepare Ni-NTA functionalized probes, silicon nitrite gold-coated cantilevers with spring constant of 0.08–0.12 N/m (OMCL-TR400PB: Olympus, Japan) were used. To immobilize the intimin onto a gold substrate (ARGOLD-15 mm: Asylum Research, CA), 10 mg/ml of purified intimin reduced in 10 mM TCEP-PBS for 16 h was placed onto the gold substrate for 30 min.

### Preparation of whole cell lysates, SDS-PAGE and western blot to compare expression levels of Strep-tagged Intimin variants

The plasmids pIBA2-Int-Strep and pIBA-Int-GS_Connector_-Strep were introduced into *E. coli* BL21 by chemical transformation. Single clones were used for a second transformation with pACYC-EGFP or pACYC-RedEx to obtain fluorescently labeled strains (originally intended to be used for adhesion assays). These plasmids are based on pACYC-184, where first a constitutive pTac-promoter via restriction sites BamHI/HindIII was inserted. The promoter was amplified from pMK4^65^ using the primers Ptac_For and Ptac_Rev. The coding sequences of EGFP (derived from pEGFP-N1) or DsRedExpress (derived from pDsRedExpress-C1) was inserted. EGFP and RedExwere amplified using the primers EGFP_For and EGFP_Rev or RedEx_For and RedEx_Rev, respectively. To compare expression levels of Int-Strep and Int-GS_Connector_-Strep, overnight cultures containing ampicillin (100 μg/ml) and chloramphenicol (25 μg/ml) in LB were inoculated with a single colony from a selective agar plate. Bacteria were grown with shaking overnight at 27 °C. The following day, the OD_600_ was measured and subcultures were inoculated at an OD 0.1 in LB with the same supplements as in the overnight culture. After 2 h of growth at 27 °C, 1 ml samples were withdrawn to prepare whole cell lysates for western blot (“before induction”). In the remaining cultures, the expression of Int-Strep and Int-GS_Connector_-Strep was induced by addition of AHTC (200 ng/ml final concentration), and the cultures were grown further for 3 h at 27 °C with shaking. After 3 h, 1 ml samples were again withdrawn and prepared for SDS-PAGE. Whole cell lysates were prepared so that 10 µl of sample finally contained 2.5 × 10^6^ cells. 10 µl samples were separated on a 4–20% TGX gradient gel (BioRad) and blotted onto nitrocellulose. The membrane was blocked overnight at 4 °C with 5% milk in TBS-T. Strep-tagged proteins were finally detected using an anti-Strep Tag II antibody (IBA Lifesciences) diluted 1:1000 in 5% milk in TBS-T for 2 h at room temperature. After two washes for 10 min each with TBS-T, the secondary antibody (goat-anti mouse HRP, Dianova), diluted 1:1000 in 5% milk in TBS-T was applied for 1 h at room temperature with gentle shaking. After two additional washing steps with TBS-T for 10 min each, detection was carried out using the Clarity Western ECL Kit (BioRad). Images were taken using a chemiluminescence imaging system (Peqlab).

### SEC-MALS

SEC-MALS experiments were performed on the Agilent 1260 Infinity II LC System (Agilent, Santa Clara, US) with Dawn Heleos-II (Wyatt Technology, Santa Barbara, USA), Agilent 1230 infinity (Agilent, Santa Clara, US) and Optilab rex 633 nm (Wyatt Technology, Santa Barbara, USA) detectors using Superdex 75 10/300 column (GE). Indicated concentrations of D00–D0 and D0–D1 were prepared in buffer (10 mM HEPES-NaOH, pH 7.5 and 100 mM KCl) and all samples were filtered with a 0.2 µm filters (Sartorius, 17,821-K) prior to measurements. 50 µl was injected per sample and ran at 0.4 ml/min. Data collection and processing was performed using ASTRA software V7.2 (Wyatt Technology, Santa Barbara, USA).

## Supplementary information

Supplementary Information 1.

Supplementary Information 2.

Supplementary Data 1.

Supplementary Data 2.

Supplementary Information 3.

## Data Availability

Data that support the findings of this study have been deposited in protein data bank with the PDB identifier 6TQD and 6TPL. Additional data are implemented as Supplementary data. All other relevant data are available from the corresponding author.

## Identifier code

6TQD, 6TPL.
