# Supplementary material for: The extracellular juncture domains in the intimin passenger adopt a constitutively extended conformation inducing restraints to its sphere of action
Source: Sci Rep. 2020 Dec 4;10:21249. doi: 10.1038/s41598-020-77706-7 (PMC7718877; doi:10.1038/s41598-020-77706-7)
Supplement: Supplementary file 1 — Supplementary Information 1. [file 41598_2020_77706_MOESM1_ESM.pdf]

Full wwPDB X-ray Structure Validation Report (i)

Dec 13, 2019 – 04:11 pm GMT

PDB ID	:	6TPL
Title	:	D0-D1 domain of Intimin
Deposited on	:	2019-12-13
Resolution	:	1.80 Å(reported)

This is a Full wwPDB X-ray Structure Validation Report.

This report is produced by the wwPDB biocuration pipeline after annotation of the structure.

We welcome your comments at *validation@mail.wwpdb.org*
A user guide is available at
https://www.wwpdb.org/validation/2017/XrayValidationReportHelp
with specific help available everywhere you see the (i) symbol.

CONFIDENTIAL VALIDATION REPORT

The following versions of software and data (see references (i)) were used in the production of this report:

MolProbity	:	4.02b-467
Xtriage (Phenix)	:	1.13
EDS	:	2.7
Percentile statistics	:	20171227.v01 (using entries in the PDB archive December 27th 2017)
Refmac	:	5.8.0158
CCP4	:	7.0.044 (Gargrove)
Ideal geometry (proteins)	:	Engh & Huber (2001)
Ideal geometry (DNA, RNA)	:	Parkinson et al. (1996)
Validation Pipeline (wwPDB-VP)	:	2.7

1 Overall quality at a glance (i)

The following experimental techniques were used to determine the structure:
X-RAY DIFFRACTION

The reported resolution of this entry is 1.80 Å.

Percentile scores (ranging between 0-100) for global validation metrics of the entry are shown in the following graphic. The table shows the number of entries on which the scores are based.

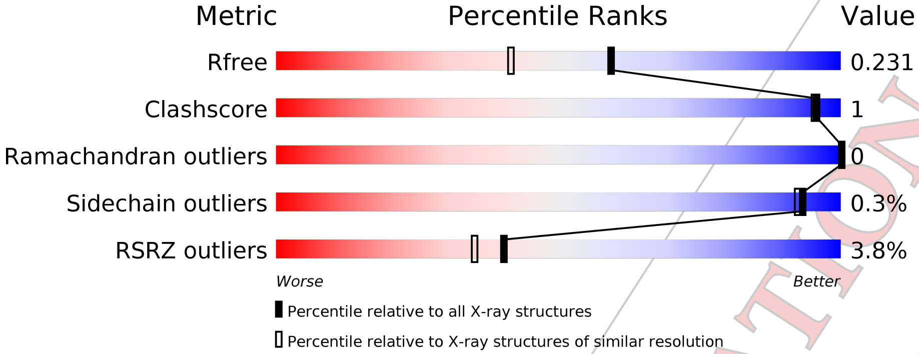

Metric	Whole archive (#Entries)	Similar resolution (#Entries, resolution range(Å))
R_{free}	111664	5253 (1.80-1.80)
Clashscore	122126	6077 (1.80-1.80)
Ramachandran outliers	120053	6011 (1.80-1.80)
Sidechain outliers	120020	6010 (1.80-1.80)
RSRZ outliers	108989	5157 (1.80-1.80)

The table below summarises the geometric issues observed across the polymeric chains and their fit to the electron density. The red, orange, yellow and green segments on the lower bar indicate the fraction of residues that contain outliers for >=3, 2, 1 and 0 types of geometric quality criteria respectively. A grey segment represents the fraction of residues that are not modelled. The numeric value for each fraction is indicated below the corresponding segment, with a dot representing fractions <=5% The upper red bar (where present) indicates the fraction of residues that have poor fit to the electron density. The numeric value is given above the bar.

Mol	Chain	Length	Quality of chain
1	A	199	95% 5% •
1	B	199	8%<br>96% • •

CONFIDENTIAL VALIDATION REPORT

2 Entry composition (i)

There are 4 unique types of molecules in this entry. The entry contains 6362 atoms, of which 2902 are hydrogens and 0 are deuteriums.

In the tables below, the ZeroOcc column contains the number of atoms modelled with zero occupancy, the AltConf column contains the number of residues with at least one atom in alternate conformation and the Trace column contains the number of residues modelled with at most 2 atoms.

- Molecule 1 is a protein called Intimin.

Mol	Chain	Residues	Atoms						ZeroOcc	AltConf	Trace
1	A	198	Total 2907	C 891	H 1465	N 240	O 309	S 2	0	3	0
1	B	195	Total 2848	C 873	H 1437	N 235	O 301	S 2	0	2	0

- Molecule 2 is MAGNESIUM ION (three-letter code: MG) (formula: Mg).

Mol	Chain	Residues	Atoms		ZeroOcc	AltConf
2	B	1	Total 1	Mg 1	0	0
2	A	3	Total 3	Mg 3	0	0

- Molecule 3 is CHLORIDE ION (three-letter code: CL) (formula: Cl).

Mol	Chain	Residues	Atoms		ZeroOcc	AltConf
3	B	1	Total 1	Cl 1	0	0
3	A	2	Total 2	Cl 2	0	0

- Molecule 4 is water.

Mol	Chain	Residues	Atoms		ZeroOcc	AltConf
4	A	365	Total 365	O 365	0	0
4	B	235	Total 235	O 235	0	0

CONFIDENTIAL VALIDATION REPORT

3 Residue-property plots (i)

These plots are drawn for all protein, RNA and DNA chains in the entry. The first graphic for a chain summarises the proportions of the various outlier classes displayed in the second graphic. The second graphic shows the sequence view annotated by issues in geometry and electron density. Residues are color-coded according to the number of geometric quality criteria for which they contain at least one outlier: green = 0, yellow = 1, orange = 2 and red = 3 or more. A red dot above a residue indicates a poor fit to the electron density (RSRZ > 2). Stretches of 2 or more consecutive residues without any outlier are shown as a green connector. Residues present in the sample, but not in the model, are shown in grey.

- Molecule 1: Intimin

Chain A:

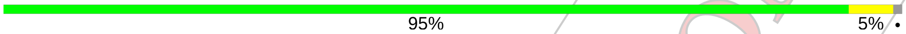

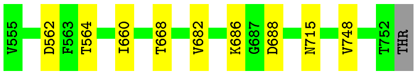

- Molecule 1: Intimin

Chain B:

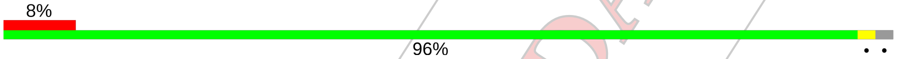

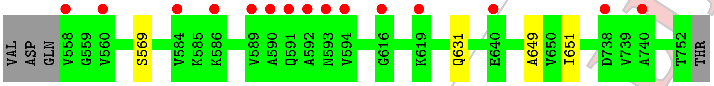

CONFIDENTIAL VALIDATION REPORT

4 Data and refinement statistics (i)

Property	Value	Source
Space group	P 1	Depositor
Cell constants<br>a, b, c, α, β, γ	33.74Å 45.33Å 67.56Å<br>97.08° 97.55° 99.90°	Depositor
Resolution (Å)	24.77 – 1.80<br>44.15 – 1.80	Depositor<br>EDS
% Data completeness<br>(in resolution range)	91.4 (24.77-1.80)<br>91.4 (44.15-1.80)	Depositor<br>EDS
R_{merge}	0.10	Depositor
R_{sym}	(Not available)	Depositor
$< I/\sigma(I) >$ [1]	1.49 (at 1.79Å)	Xtriage
Refinement program	PHENIX 1.16_3549, PHENIX 1.16_3549	Depositor
R, R_{free}	0.187 , 0.231<br>0.188 , 0.231	Depositor<br>DCC
R_{free} test set	1617 reflections (4.91%)	wwPDB-VP
Wilson B-factor (Å^2)	15.0	Xtriage
Anisotropy	0.843	Xtriage
Bulk solvent k_{sol}(e/Å^3), B_{sol}(Å^2)	0.36 , 48.3	EDS
L-test for twinning[2]	$< \|L\| > = 0.51$, $< L^2 > = 0.34$	Xtriage
Estimated twinning fraction	No twinning to report.	Xtriage
F_o,F_c correlation	0.94	EDS
Total number of atoms	6362	wwPDB-VP
Average B, all atoms (Å^2)	27.0	wwPDB-VP

Xtriage's analysis on translational NCS is as follows: *The largest off-origin peak in the Patterson function is 10.62% of the height of the origin peak. No significant pseudotranslation is detected.*

[1] Intensities estimated from amplitudes.

[2] Theoretical values of $< |L| >$, $< L^2 >$ for acentric reflections are 0.5, 0.333 respectively for untwinned datasets, and 0.375, 0.2 for perfectly twinned datasets.

CONFIDENTIAL VALIDATION REPORT

5 Model quality (i)

5.1 Standard geometry (i)

Bond lengths and bond angles in the following residue types are not validated in this section: MG, CL

The Z score for a bond length (or angle) is the number of standard deviations the observed value is removed from the expected value. A bond length (or angle) with $|Z| > 5$ is considered an outlier worth inspection. RMSZ is the root-mean-square of all Z scores of the bond lengths (or angles).

Mol	Chain	Bond lengths		Bond angles	
		RMSZ	$\#\|Z\| > 5$	RMSZ	$\#\|Z\| > 5$
1	A	0.28	0/1464	0.53	0/1994
1	B	0.27	0/1429	0.51	0/1946
All	All	0.27	0/2893	0.52	0/3940

There are no bond length outliers.

There are no bond angle outliers.

There are no chirality outliers.

There are no planarity outliers.

5.2 Too-close contacts (i)

In the following table, the Non-H and H(model) columns list the number of non-hydrogen atoms and hydrogen atoms in the chain respectively. The H(added) column lists the number of hydrogen atoms added and optimized by MolProbity. The Clashes column lists the number of clashes within the asymmetric unit, whereas Symm-Clashes lists symmetry related clashes.

Mol	Chain	Non-H	H(model)	H(added)	Clashes	Symm-Clashes
1	A	1442	1465	1458	5	0
1	B	1411	1437	1431	2	0
2	A	3	0	0	0	0
2	B	1	0	0	0	0
3	A	2	0	0	0	0
3	B	1	0	0	0	0
4	A	365	0	0	2	1
4	B	235	0	0	0	1
All	All	3460	2902	2889	7	1

The all-atom clashscore is defined as the number of clashes found per 1000 atoms (including hydrogen atoms). The all-atom clashscore for this structure is 1.

CONFIDENTIAL VALIDATION REPORT

All (7) close contacts within the same asymmetric unit are listed below, sorted by their clash magnitude.

Atom-1	Atom-2	Interatomic distance (Å)	Clash overlap (Å)
1:A:715:ASN:OD1	4:A:901:HOH:O	2.17	0.58
1:A:562:ASP:OD1	1:A:564:THR:HG23	2.09	0.53
1:A:660:ILE:HG23	1:A:682:VAL:HG13	1.96	0.48
1:B:631:GLN:HG2	1:B:649:ALA:HB1	1.96	0.48
1:B:569:SER:HA	1:B:651:ILE:O	2.20	0.41
1:A:686:LYS:NZ	4:A:921:HOH:O	2.53	0.40
1:A:668:THR:HA	1:A:748:VAL:HG12	2.03	0.40

All (1) symmetry-related close contacts are listed below. The label for Atom-2 includes the symmetry operator and encoded unit-cell translations to be applied.

Atom-1	Atom-2	Interatomic distance (Å)	Clash overlap (Å)
4:A:1082:HOH:O	4:B:1085:HOH:O[1_444]	2.15	0.05

5.3 Torsion angles ⓘ

5.3.1 Protein backbone ⓘ

In the following table, the Percentiles column shows the percent Ramachandran outliers of the chain as a percentile score with respect to all X-ray entries followed by that with respect to entries of similar resolution.

The Analysed column shows the number of residues for which the backbone conformation was analysed, and the total number of residues.

Mol	Chain	Analysed	Favoured	Allowed	Outliers	Percentiles	
1	A	199/199 (100%)	196 (98%)	3 (2%)	0	100	100
1	B	195/199 (98%)	189 (97%)	6 (3%)	0	100	100
All	All	394/398 (99%)	385 (98%)	9 (2%)	0	100	100

There are no Ramachandran outliers to report.

5.3.2 Protein sidechains ⓘ

In the following table, the Percentiles column shows the percent sidechain outliers of the chain as a percentile score with respect to all X-ray entries followed by that with respect to entries of similar resolution.

CONFIDENTIAL VALIDATION REPORT

The Analysed column shows the number of residues for which the sidechain conformation was analysed, and the total number of residues.

Mol	Chain	Analysed	Rotameric	Outliers	Percentiles	
1	A	164/162 (101%)	163 (99%)	1 (1%)	87	86
1	B	159/162 (98%)	159 (100%)	0	100	100
All	All	323/324 (100%)	322 (100%)	1 (0%)	93	92

All (1) residues with a non-rotameric sidechain are listed below:

Mol	Chain	Res	Type
1	A	688	ASP

Some sidechains can be flipped to improve hydrogen bonding and reduce clashes. All (2) such sidechains are listed below:

Mol	Chain	Res	Type
1	A	715	ASN
1	B	648	ASN

5.3.3 RNA (i)

There are no RNA molecules in this entry.

5.4 Non-standard residues in protein, DNA, RNA chains (i)

There are no non-standard protein/DNA/RNA residues in this entry.

5.5 Carbohydrates (i)

There are no carbohydrates in this entry.

5.6 Ligand geometry (i)

Of 7 ligands modelled in this entry, 7 are monoatomic - leaving 0 for Mogul analysis.

There are no bond length outliers.

There are no bond angle outliers.

There are no chirality outliers.

There are no torsion outliers.

CONFIDENTIAL VALIDATION REPORT

There are no ring outliers.

No monomer is involved in short contacts.

5.7 Other polymers (i)

There are no such residues in this entry.

5.8 Polymer linkage issues (i)

There are no chain breaks in this entry.

CONFIDENTIAL VALIDATION REPORT

6 Fit of model and data (i)

6.1 Protein, DNA and RNA chains (i)

In the following table, the column labelled '#RSRZ> 2' contains the number (and percentage) of RSRZ outliers, followed by percent RSRZ outliers for the chain as percentile scores relative to all X-ray entries and entries of similar resolution. The OWAB column contains the minimum, median, 95th percentile and maximum values of the occupancy-weighted average B-factor per residue. The column labelled 'Q< 0.9' lists the number of (and percentage) of residues with an average occupancy less than 0.9.

Mol	Chain	Analysed	<RSRZ>	#RSRZ>2			OWAB(Å^2)	Q<0.9
1	A	198/199 (99%)	-0.30	0	100	100	10, 17, 34, 53	0
1	B	195/199 (97%)	0.24	15 (7%)	13	10	9, 26, 57, 74	0
All	All	393/398 (98%)	-0.04	15 (3%)	40	35	9, 20, 51, 74	0

All (15) RSRZ outliers are listed below:

Mol	Chain	Res	Type	RSRZ
1	B	584	VAL	4.7
1	B	591	GLN	4.2
1	B	616	GLY	4.0
1	B	640	GLU	3.5
1	B	558	VAL	3.2
1	B	592	ALA	3.0
1	B	586	LYS	2.8
1	B	738	ASP	2.8
1	B	593	ASN	2.5
1	B	560	VAL	2.4
1	B	589	VAL	2.4
1	B	619	LYS	2.3
1	B	594	VAL	2.2
1	B	740	ALA	2.1
1	B	590	ALA	2.1

6.2 Non-standard residues in protein, DNA, RNA chains (i)

There are no non-standard protein/DNA/RNA residues in this entry.

6.3 Carbohydrates (i)

There are no carbohydrates in this entry.

CONFIDENTIAL VALIDATION REPORT

6.4 Ligands (i)

In the following table, the Atoms column lists the number of modelled atoms in the group and the number defined in the chemical component dictionary. The B-factors column lists the minimum, median, 95th percentile and maximum values of B factors of atoms in the group. The column labelled 'Q< 0.9' lists the number of atoms with occupancy less than 0.9.

Mol	Type	Chain	Res	Atoms	RSCC	RSR	B-factors($Å^2$)	Q<0.9
2	MG	B	801	1/1	0.94	0.11	31,31,31,31	0
2	MG	A	803	1/1	0.95	0.16	49,49,49,49	0
2	MG	A	802	1/1	0.95	0.12	29,29,29,29	0
3	CL	B	802	1/1	0.99	0.04	24,24,24,24	0
3	CL	A	805	1/1	0.99	0.04	23,23,23,23	0
3	CL	A	804	1/1	0.99	0.06	21,21,21,21	0
2	MG	A	801	1/1	0.99	0.12	24,24,24,24	0

6.5 Other polymers (i)

There are no such residues in this entry.

CONFIDENTIAL VALIDATION REPORT
