# Supplementary material for: The extracellular juncture domains in the intimin passenger adopt a constitutively extended conformation inducing restraints to its sphere of action
Source: Sci Rep. 2020 Dec 4;10:21249. doi: 10.1038/s41598-020-77706-7 (PMC7718877; doi:10.1038/s41598-020-77706-7)
Supplement: Supplementary file 2 — Supplementary Information 2. [file 41598_2020_77706_MOESM2_ESM.pdf]

Full wwPDB X-ray Structure Validation Report (i)

Dec 17, 2019 – 09:47 am GMT

PDB ID	:	6TQD
Title	:	D00-D0 domain of Intimin
Deposited on	:	2019-12-16
Resolution	:	1.48 Å(reported)

This is a Full wwPDB X-ray Structure Validation Report.

This report is produced by the wwPDB biocuration pipeline after annotation of the structure.

We welcome your comments at *validation@mail.wwpdb.org*
A user guide is available at
https://www.wwpdb.org/validation/2017/XrayValidationReportHelp
with specific help available everywhere you see the (i) symbol.

CONFIDENTIAL VALIDATION REPORT

The following versions of software and data (see references (i)) were used in the production of this report:

MolProbity	:	4.02b-467
Mogul	:	1.8.0 (224370), CSD as540be (2019)
Xtriage (Phenix)	:	1.13
EDS	:	2.7
Percentile statistics	:	20171227.v01 (using entries in the PDB archive December 27th 2017)
Refmac	:	5.8.0158
CCP4	:	7.0.044 (Gargrove)
Ideal geometry (proteins)	:	Engh & Huber (2001)
Ideal geometry (DNA, RNA)	:	Parkinson et al. (1996)
Validation Pipeline (wwPDB-VP)	:	2.7

1 Overall quality at a glance (i)

The following experimental techniques were used to determine the structure:
X-RAY DIFFRACTION

The reported resolution of this entry is 1.48 Å.

Percentile scores (ranging between 0-100) for global validation metrics of the entry are shown in the following graphic. The table shows the number of entries on which the scores are based.

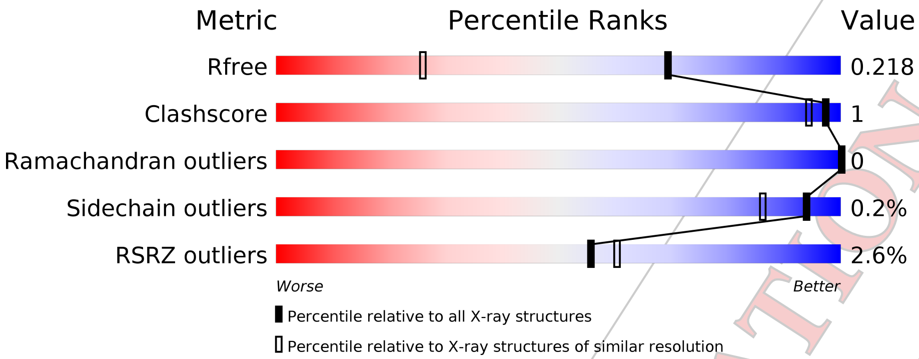

Metric	Whole archive (#Entries)	Similar resolution (#Entries, resolution range(Å))
R_{free}	111664	3964 (1.50-1.46)
Clashscore	122126	4202 (1.50-1.46)
Ramachandran outliers	120053	4117 (1.50-1.46)
Sidechain outliers	120020	4115 (1.50-1.46)
RSRZ outliers	108989	3889 (1.50-1.46)

The table below summarises the geometric issues observed across the polymeric chains and their fit to the electron density. The red, orange, yellow and green segments on the lower bar indicate the fraction of residues that contain outliers for >=3, 2, 1 and 0 types of geometric quality criteria respectively. A grey segment represents the fraction of residues that are not modelled. The numeric value for each fraction is indicated below the corresponding segment, with a dot representing fractions <=5% The upper red bar (where present) indicates the fraction of residues that have poor fit to the electron density. The numeric value is given above the bar.

Mol	Chain	Length	Quality of chain
1	A	207	2% 97% ••
1	B	207	2% 99% •
1	C	207	3% 97% ••
1	D	207	2% 97% •
1	E	207	% 94% • •
1	F	207	4% 99% •

CONFIDENTIAL VALIDATION REPORT

2 Entry composition (i)

There are 6 unique types of molecules in this entry. The entry contains 20690 atoms, of which 9316 are hydrogens and 0 are deuteriums.

In the tables below, the ZeroOcc column contains the number of atoms modelled with zero occupancy, the AltConf column contains the number of residues with at least one atom in alternate conformation and the Trace column contains the number of residues modelled with at most 2 atoms.

- Molecule 1 is a protein called Intimin.

Mol	Chain	Residues	Atoms						ZeroOcc	AltConf	Trace
			Total	C	H	N	O	S			
1	A	204	3190	975	1597	284	333	1	0	23	0
1	B	206	3134	960	1567	280	326	1	0	12	0
1	C	204	3018	928	1507	268	314	1	0	2	0
1	D	206	3100	950	1549	277	323	1	0	9	0
1	E	202	3087	947	1543	275	321	1	0	16	0
1	F	205	3072	942	1535	275	319	1	0	6	0

There are 6 discrepancies between the modelled and reference sequences:

Chain	Residue	Modelled	Actual	Comment	Reference
A	449	SER	-	expression tag	UNP P19809
B	449	SER	-	expression tag	UNP P19809
C	449	SER	-	expression tag	UNP P19809
D	449	SER	-	expression tag	UNP P19809
E	449	SER	-	expression tag	UNP P19809
F	449	SER	-	expression tag	UNP P19809

- Molecule 2 is BROMIDE ION (three-letter code: BR) (formula: Br).

Mol	Chain	Residues	Atoms		ZeroOcc	AltConf
			Total	Br		
2	D	1	1	1	0	0
2	E	1	1	1	0	0
2	B	2	2	2	0	0

Continued on next page...

CONFIDENTIAL VALIDATION REPORT

Continued from previous page...

Mol	Chain	Residues	Atoms	ZeroOcc	AltConf
2	C	2	Total Br<br>2 2	0	0
2	A	2	Total Br<br>2 2	0	0
2	F	1	Total Br<br>1 1	0	0

- Molecule 3 is SODIUM ION (three-letter code: NA) (formula: Na).

Mol	Chain	Residues	Atoms	ZeroOcc	AltConf
3	B	1	Total Na<br>1 1	0	0
3	A	1	Total Na<br>1 1	0	0

- Molecule 4 is CHLORIDE ION (three-letter code: CL) (formula: Cl).

Mol	Chain	Residues	Atoms	ZeroOcc	AltConf
4	B	1	Total Cl<br>1 1	0	0
4	A	3	Total Cl<br>3 3	0	0
4	D	1	Total Cl<br>1 1	0	0
4	C	1	Total Cl<br>1 1	0	0

- Molecule 5 is TETRAETHYLENE GLYCOL (three-letter code: PG4) (formula: $C_8H_{18}O_5$).

CONFIDENTIAL VALIDATION REPORT

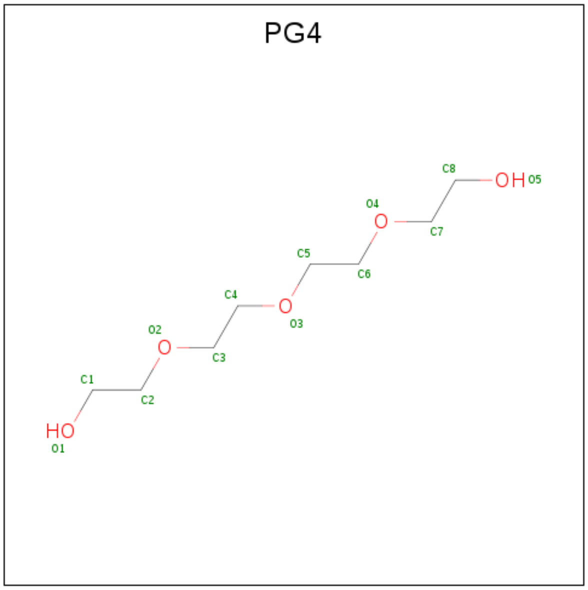

Mol	Chain	Residues	Atoms	ZeroOcc	AltConf
5	D	1	Total C H O<br>31 8 18 5	0	0

- Molecule 6 is water.

Mol	Chain	Residues	Atoms	ZeroOcc	AltConf
6	A	344	Total O<br>344 344	0	0
6	B	340	Total O<br>340 340	0	0
6	C	327	Total O<br>327 327	0	0
6	D	375	Total O<br>376 376	0	1
6	E	326	Total O<br>327 327	0	1
6	F	327	Total O<br>327 327	0	0

CONFIDENTIAL VALIDATION REPORT

3 Residue-property plots (i)

These plots are drawn for all protein, RNA and DNA chains in the entry. The first graphic for a chain summarises the proportions of the various outlier classes displayed in the second graphic. The second graphic shows the sequence view annotated by issues in geometry and electron density. Residues are color-coded according to the number of geometric quality criteria for which they contain at least one outlier: green = 0, yellow = 1, orange = 2 and red = 3 or more. A red dot above a residue indicates a poor fit to the electron density (RSRZ > 2). Stretches of 2 or more consecutive residues without any outlier are shown as a green connector. Residues present in the sample, but not in the model, are shown in grey.

• Molecule 1: Intimin

Chain A:
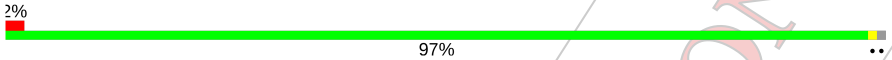

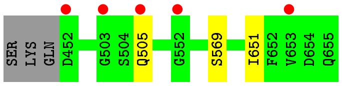

• Molecule 1: Intimin

Chain B:
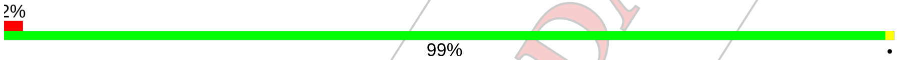

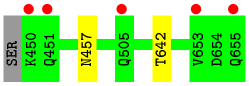

• Molecule 1: Intimin

Chain C:
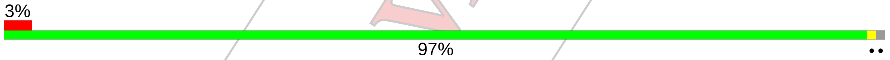

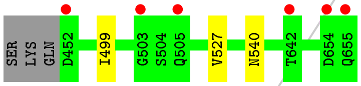

• Molecule 1: Intimin

Chain D:
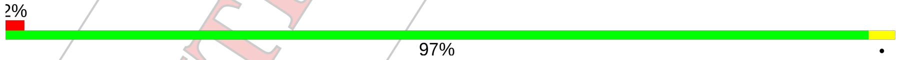

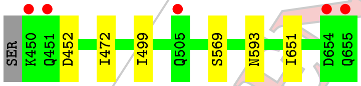

• Molecule 1: Intimin

Chain E:
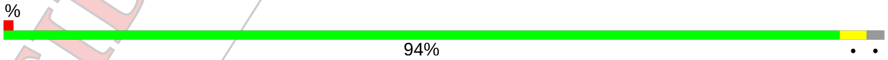

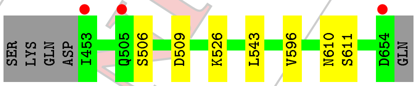

• Molecule 1: Intimin

CONFIDENTIAL VALIDATION REPORT

Chain F:

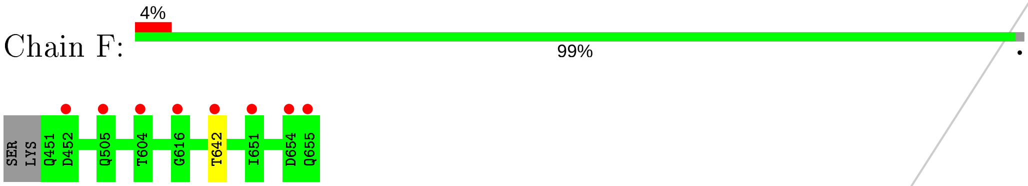

CONFIDENTIAL VALIDATION REPORT

4 Data and refinement statistics (i)

Property	Value	Source
Space group	P 1	Depositor
Cell constants<br>a, b, c, α, β, γ	56.04Å 65.47Å 79.38Å<br>77.78° 76.55° 84.76°	Depositor
Resolution (Å)	24.83 – 1.48<br>63.93 – 1.48	Depositor<br>EDS
% Data completeness<br>(in resolution range)	98.2 (24.83-1.48)<br>98.3 (63.93-1.48)	Depositor<br>EDS
R_{merge}	0.13	Depositor
R_{sym}	(Not available)	Depositor
$< I/\sigma(I) >$ [1]	1.63 (at 1.48Å)	Xtriage
Refinement program	PHENIX 1.16_3549, PHENIX 1.16_3549	Depositor
R, R_{free}	0.179 , 0.215<br>0.182 , 0.218	Depositor<br>DCC
R_{free} test set	8672 reflections (4.93%)	wwPDB-VP
Wilson B-factor (Å^2)	9.5	Xtriage
Anisotropy	0.577	Xtriage
Bulk solvent k_{sol}(e/Å^3), B_{sol}(Å^2)	0.36 , 42.8	EDS
L-test for twinning[2]	$< \|L\| > = 0.50$, $< L^2 > = 0.33$	Xtriage
Estimated twinning fraction	No twinning to report.	Xtriage
F_o,F_c correlation	0.96	EDS
Total number of atoms	20690	wwPDB-VP
Average B, all atoms (Å^2)	19.0	wwPDB-VP

Xtriage's analysis on translational NCS is as follows: *The analyses of the Patterson function reveals a significant off-origin peak that is 60.37 % of the origin peak, indicating pseudo-translational symmetry. The chance of finding a peak of this or larger height randomly in a structure without pseudo-translational symmetry is equal to 1.5364e-05. The detected translational NCS is most likely also responsible for the elevated intensity ratio.*

[1] Intensities estimated from amplitudes.
[2] Theoretical values of $< |L| >$, $< L^2 >$ for acentric reflections are 0.5, 0.333 respectively for untwinned datasets, and 0.375, 0.2 for perfectly twinned datasets.

CONFIDENTIAL VALIDATION REPORT

5 Model quality (i)

5.1 Standard geometry (i)

Bond lengths and bond angles in the following residue types are not validated in this section: NA, PG4, BR, CL

The Z score for a bond length (or angle) is the number of standard deviations the observed value is removed from the expected value. A bond length (or angle) with $|Z| > 5$ is considered an outlier worth inspection. RMSZ is the root-mean-square of all Z scores of the bond lengths (or angles).

Mol	Chain	Bond lengths		Bond angles	
		RMSZ	$\#\|Z\| > 5$	RMSZ	$\#\|Z\| > 5$
1	A	0.35	0/1706	0.58	0/2322
1	B	0.34	0/1628	0.57	0/2213
1	C	0.32	0/1533	0.57	0/2084
1	D	0.34	0/1595	0.57	0/2168
1	E	0.32	0/1633	0.57	0/2225
1	F	0.32	0/1577	0.56	0/2142
All	All	0.33	0/9672	0.57	0/13154

There are no bond length outliers.

There are no bond angle outliers.

There are no chirality outliers.

There are no planarity outliers.

5.2 Too-close contacts (i)

In the following table, the Non-H and H(model) columns list the number of non-hydrogen atoms and hydrogen atoms in the chain respectively. The H(added) column lists the number of hydrogen atoms added and optimized by MolProbity. The Clashes column lists the number of clashes within the asymmetric unit, whereas Symm-Clashes lists symmetry related clashes.

Mol	Chain	Non-H	H(model)	H(added)	Clashes	Symm-Clashes
1	A	1593	1597	1498	4	0
1	B	1567	1567	1515	3	0
1	C	1511	1507	1501	2	0
1	D	1551	1549	1513	3	0
1	E	1544	1543	1472	5	0
1	F	1537	1535	1513	3	0
2	A	2	0	0	0	0

Continued on next page...

CONFIDENTIAL VALIDATION REPORT

Continued from previous page...

Mol	Chain	Non-H	H(model)	H(added)	Clashes	Symm-Clashes
2	B	2	0	0	0	0
2	C	2	0	0	0	0
2	D	1	0	0	0	0
2	E	1	0	0	0	0
2	F	1	0	0	0	0
3	A	1	0	0	0	0
3	B	1	0	0	0	0
4	A	3	0	0	1	0
4	B	1	0	0	0	0
4	C	1	0	0	0	0
4	D	1	0	0	0	0
5	D	13	18	18	0	0
6	A	344	0	0	1	0
6	B	340	0	0	0	0
6	C	327	0	0	1	0
6	D	376	0	0	1	0
6	E	327	0	0	1	0
6	F	327	0	0	0	0
All	All	11374	9316	9030	16	0

The all-atom clashscore is defined as the number of clashes found per 1000 atoms (including hydrogen atoms). The all-atom clashscore for this structure is 1.

All (16) close contacts within the same asymmetric unit are listed below, sorted by their clash magnitude.

Atom-1	Atom-2	Interatomic distance (Å)	Clash overlap (Å)
1:A:505:GLN:HB2	1:F:642:THR:HG21	1.64	0.78
1:E:509[A]:ASP:OD1	6:E:801[A]:HOH:O	2.03	0.75
4:A:706:CL:CL	6:A:886:HOH:O	2.43	0.72
1:A:505:GLN:CB	1:F:642:THR:HG21	2.20	0.71
1:B:642:THR:HG21	1:E:506:SER:OG	1.95	0.66
1:E:526:LYS:HB3	1:E:543:LEU:HD11	1.90	0.53
1:D:593[A]:ASN:ND2	6:D:806:HOH:O	2.45	0.49
1:D:472:ILE:HD11	1:D:499:ILE:HD12	1.95	0.48
1:C:499:ILE:HD11	1:C:527:VAL:HG21	1.97	0.46
1:C:540:ASN:ND2	6:C:812:HOH:O	2.51	0.42
1:D:569:SER:HA	1:D:651:ILE:O	2.20	0.41
1:A:569:SER:HA	1:A:651:ILE:O	2.20	0.41
1:A:505:GLN:HB3	1:F:642:THR:HG21	1.97	0.41
1:E:596:VAL:O	1:E:611[A]:SER:HA	2.21	0.41
1:B:642:THR:HG21	1:E:506:SER:CB	2.50	0.40

CONFIDENTIAL VALIDATION REPORT

There are no symmetry-related clashes.

5.3 Torsion angles (i)

5.3.1 Protein backbone (i)

In the following table, the Percentiles column shows the percent Ramachandran outliers of the chain as a percentile score with respect to all X-ray entries followed by that with respect to entries of similar resolution.

The Analysed column shows the number of residues for which the backbone conformation was analysed, and the total number of residues.

Mol	Chain	Analysed	Favoured	Allowed	Outliers	Percentiles	
1	A	225/207 (109%)	224 (100%)	1 (0%)	0	100	100
1	B	216/207 (104%)	214 (99%)	2 (1%)	0	100	100
1	C	204/207 (99%)	203 (100%)	1 (0%)	0	100	100
1	D	213/207 (103%)	210 (99%)	3 (1%)	0	100	100
1	E	216/207 (104%)	214 (99%)	2 (1%)	0	100	100
1	F	209/207 (101%)	208 (100%)	1 (0%)	0	100	100
All	All	1283/1242 (103%)	1273 (99%)	10 (1%)	0	100	100

There are no Ramachandran outliers to report.

5.3.2 Protein sidechains (i)

In the following table, the Percentiles column shows the percent sidechain outliers of the chain as a percentile score with respect to all X-ray entries followed by that with respect to entries of similar resolution.

The Analysed column shows the number of residues for which the sidechain conformation was analysed, and the total number of residues.

Mol	Chain	Analysed	Rotameric	Outliers	Percentiles	
1	A	187/169 (111%)	187 (100%)	0	100	100
1	B	179/169 (106%)	179 (100%)	0	100	100
1	C	168/169 (99%)	168 (100%)	0	100	100
1	D	174/169 (103%)	173 (99%)	1 (1%)	87	74
1	E	178/169 (105%)	176 (99%)	2 (1%)	76	53
1	F	173/169 (102%)	173 (100%)	0	100	100

Continued on next page...

CONFIDENTIAL VALIDATION REPORT

Continued from previous page...

Mol	Chain	Analysed	Rotameric	Outliers	Percentiles	
All	All	1059/1014 (104%)	1056 (100%)	3 (0%)	94	85

All (3) residues with a non-rotameric sidechain are listed below:

Mol	Chain	Res	Type
1	D	452	ASP
1	E	610[A]	ASN
1	E	610[B]	ASN

Some sidechains can be flipped to improve hydrogen bonding and reduce clashes. There are no such sidechains identified.

5.3.3 RNA ⓘ

There are no RNA molecules in this entry.

5.4 Non-standard residues in protein, DNA, RNA chains ⓘ

There are no non-standard protein/DNA/RNA residues in this entry.

5.5 Carbohydrates ⓘ

There are no carbohydrates in this entry.

5.6 Ligand geometry ⓘ

Of 18 ligands modelled in this entry, 17 are monoatomic - leaving 1 for Mogul analysis.

In the following table, the Counts columns list the number of bonds (or angles) for which Mogul statistics could be retrieved, the number of bonds (or angles) that are observed in the model and the number of bonds (or angles) that are defined in the Chemical Component Dictionary. The Link column lists molecule types, if any, to which the group is linked. The Z score for a bond length (or angle) is the number of standard deviations the observed value is removed from the expected value. A bond length (or angle) with $|Z| > 2$ is considered an outlier worth inspection. RMSZ is the root-mean-square of all Z scores of the bond lengths (or angles).

Mol	Type	Chain	Res	Link	Bond lengths			Bond angles		
					Counts	RMSZ	$\#\|Z\| > 2$	Counts	RMSZ	$\#\|Z\| > 2$
5	PG4	D	701	-	12,12,12	0.54	0	11,11,11	0.49	0

CONFIDENTIAL VALIDATION REPORT

In the following table, the Chirals column lists the number of chiral outliers, the number of chiral centers analysed, the number of these observed in the model and the number defined in the Chemical Component Dictionary. Similar counts are reported in the Torsion and Rings columns. '-' means no outliers of that kind were identified.

Mol	Type	Chain	Res	Link	Chirals	Torsions	Rings
5	PG4	D	701	-	-	7/10/10/10	-

There are no bond length outliers.

There are no bond angle outliers.

There are no chirality outliers.

All (7) torsion outliers are listed below:

Mol	Chain	Res	Type	Atoms
5	D	701	PG4	O1-C1-C2-O2
5	D	701	PG4	O3-C5-C6-O4
5	D	701	PG4	C4-C3-O2-C2
5	D	701	PG4	C3-C4-O3-C5
5	D	701	PG4	C1-C2-O2-C3
5	D	701	PG4	O2-C3-C4-O3
5	D	701	PG4	C6-C5-O3-C4

There are no ring outliers.

No monomer is involved in short contacts.

5.7 Other polymers (i)

There are no such residues in this entry.

5.8 Polymer linkage issues (i)

There are no chain breaks in this entry.

CONFIDENTIAL VALIDATION REPORT

6 Fit of model and data (i)

6.1 Protein, DNA and RNA chains (i)

In the following table, the column labelled '#RSRZ> 2' contains the number (and percentage) of RSRZ outliers, followed by percent RSRZ outliers for the chain as percentile scores relative to all X-ray entries and entries of similar resolution. The OWAB column contains the minimum, median, 95th percentile and maximum values of the occupancy-weighted average B-factor per residue. The column labelled 'Q< 0.9' lists the number of (and percentage) of residues with an average occupancy less than 0.9.

Mol	Chain	Analysed	<RSRZ>	#RSRZ>2			OWAB(Å^2)	Q<0.9
1	A	204/207 (98%)	-0.10	5 (2%)	57	62	7, 14, 29, 50	0
1	B	206/207 (99%)	-0.08	5 (2%)	59	63	6, 13, 27, 53	0
1	C	204/207 (98%)	0.03	6 (2%)	51	56	7, 15, 28, 75	0
1	D	206/207 (99%)	-0.12	5 (2%)	59	63	7, 13, 31, 92	0
1	E	202/207 (97%)	0.02	3 (1%)	73	77	8, 16, 30, 70	0
1	F	205/207 (99%)	0.01	8 (3%)	39	43	6, 16, 32, 65	0
All	All	1227/1242 (98%)	-0.04	32 (2%)	56	60	6, 15, 30, 92	0

All (32) RSRZ outliers are listed below:

Mol	Chain	Res	Type	RSRZ
1	C	505	GLN	5.7
1	D	505	GLN	5.1
1	F	655	GLN	4.8
1	D	451	GLN	4.7
1	E	654	ASP	3.9
1	B	450	LYS	3.7
1	A	505	GLN	3.7
1	F	642	THR	3.5
1	C	452	ASP	3.5
1	E	505	GLN	3.4
1	C	642	THR	3.4
1	F	654	ASP	3.0
1	C	655	GLN	3.0
1	B	653	VAL	2.9
1	C	654	ASP	2.9
1	B	451	GLN	2.8
1	D	655	GLN	2.8
1	F	505	GLN	2.6
1	C	503	GLY	2.6

Continued on next page...

CONFIDENTIAL VALIDATION REPORT

Continued from previous page...

Mol	Chain	Res	Type	RSRZ
1	A	552	GLY	2.5
1	F	651	ILE	2.5
1	E	453	ILE	2.5
1	F	616	GLY	2.5
1	A	503	GLY	2.4
1	B	505	GLN	2.4
1	A	653	VAL	2.3
1	F	452	ASP	2.3
1	A	452	ASP	2.3
1	D	450	LYS	2.2
1	D	654	ASP	2.2
1	F	604	THR	2.2
1	B	655	GLN	2.0

6.2 Non-standard residues in protein, DNA, RNA chains (i)

There are no non-standard protein/DNA/RNA residues in this entry.

6.3 Carbohydrates (i)

There are no carbohydrates in this entry.

6.4 Ligands (i)

In the following table, the Atoms column lists the number of modelled atoms in the group and the number defined in the chemical component dictionary. The B-factors column lists the minimum, median, 95th percentile and maximum values of B factors of atoms in the group. The column labelled 'Q< 0.9' lists the number of atoms with occupancy less than 0.9.

Mol	Type	Chain	Res	Atoms	RSCC	RSR	B-factors($Å^2$)	Q<0.9
5	PG4	D	701	13/13	0.83	0.34	16,38,45,47	0
4	CL	C	703	1/1	0.90	0.11	31,31,31,31	0
4	CL	A	705	1/1	0.91	0.22	35,35,35,35	0
2	BR	F	701	1/1	0.93	0.13	57,57,57,57	0
3	NA	B	703	1/1	0.94	0.18	29,29,29,29	0
3	NA	A	703	1/1	0.96	0.10	23,23,23,23	0
2	BR	B	701	1/1	0.96	0.08	19,19,19,19	1
2	BR	B	702	1/1	0.97	0.07	16,16,16,16	1
4	CL	A	704	1/1	0.97	0.13	23,23,23,23	0
2	BR	C	702	1/1	0.97	0.09	48,48,48,48	1

Continued on next page...

CONFIDENTIAL VALIDATION REPORT

Continued from previous page...

Mol	Type	Chain	Res	Atoms	RSCC	RSR	B-factors($Å^2$)	Q<0.9
4	CL	D	703	1/1	0.98	0.04	23,23,23,23	0
2	BR	A	702	1/1	0.98	0.06	19,19,19,19	1
4	CL	A	706	1/1	0.98	0.12	23,23,23,23	0
2	BR	E	701	1/1	0.99	0.06	16,16,16,16	1
2	BR	C	701	1/1	0.99	0.03	24,24,24,24	1
4	CL	B	704	1/1	0.99	0.05	23,23,23,23	0
2	BR	A	701	1/1	0.99	0.07	19,19,19,19	1
2	BR	D	702	1/1	0.99	0.05	17,17,17,17	1

6.5 Other polymers (i)

There are no such residues in this entry.

CONFIDENTIAL VALIDATION REPORT
