# Supplementary material for: The extracellular juncture domains in the intimin passenger adopt a constitutively extended conformation inducing restraints to its sphere of action
Source: Sci Rep. 2020 Dec 4;10:21249. doi: 10.1038/s41598-020-77706-7 (PMC7718877; doi:10.1038/s41598-020-77706-7)
Supplement: Supplementary file 4 — Supplementary Data 2. [file 41598_2020_77706_MOESM4_ESM.html]

supp\_data\_1


In [1]:

```
import numpy as np
import matplotlib as mpl
import matplotlib.pyplot as plt
%matplotlib inline
from jinja2 import Template
import mdtraj as md
try:
    workdir
except NameError:
    workdir=%pwd
else:
    %cd $workdir
```

### Download and compile Faunus¶

In [ ]:

```
%%bash -s "$workdir"
git clone https://github.com/mlund/faunus.git 
cd faunus
git checkout v2.4.0
cmake . -DENABLE_OPENMP=OFF -DCMAKE_BUILD_TYPE=Release
make -j4 faunus
cd $1
```

### Faunus input file¶

In [ ]:

```
template = Template("""comment: "Intimin."
temperature: {{T}}
random: {seed: hardware}
geometry: {type: slit, length: {{[L,L,L]}}}
mcloop: {macro: 100, micro: {{micro}}}

atomlist:
    - B: {sigma: 50, eps: 1, dp: 0}
    - D00: {sigma: 40, eps: 1, dp: 0}
    - L1: {sigma: 10, eps: 1, dp: 3}
    - D0: {sigma: 40, eps: 1, dp: 3}
    - L2: {sigma: 10, eps: 1, dp: 3}
    - D1: {sigma: 40, eps: 1, dp: 3}
    - L3: {sigma: 10, eps: 1, dp: 3}
    - D2: {sigma: 40, eps: 1, dp: 3}
    - L4: {sigma: 10, eps: 1, dp: 3}
    - D3: {sigma: 40, eps: 1, dp: 3}
    
moleculelist:
    - intimin:
        structure:
            - B: [0,0,{{5-L*0.5}}]
            - D00: [0,0,{{50-L*0.5}}]    
            - L1: [0,0,{{76-L*0.5}}]    
            - D0: [0,0,{{98-L*0.5}}]    
            - L2: [0,0,{{120-L*0.5}}]    
            - D1: [0,0,{{142-L*0.5}}]    
            - L3: [0,0,{{164-L*0.5}}]    
            - D2: [0,0,{{186-L*0.5}}] 
            - L4: [0,0,{{212-L*0.5}}]
            - D3: [0,0,{{238-L*0.5}}]
        bondlist:
            - harmonic: { index: [1,2], k: 1, req: 26 }
            - harmonic: { index: [2,3], k: 0.5, req: 21 }
            - harmonic: { index: [3,4], k: 0.5, req: 21 }
            - harmonic: { index: [4,5], k: 0.5, req: 21 }
            - harmonic: { index: [5,6], k: 0.5, req: 21 }
            - harmonic: { index: [6,7], k: 0.5, req: 21 }
            - harmonic: { index: [7,8], k: 0.5, req: 21 }
            - harmonic: { index: [8,9], k: 0.5, req: 21 }
            - harmonic_torsion: { index: [1,2,3], k: {{k}}, aeq: {{aeq}} }
            - harmonic_torsion: { index: [2,3,4], k: 30, aeq: 150 }
            - harmonic_torsion: { index: [3,4,5], k: 1, aeq: 110 }
            - harmonic_torsion: { index: [4,5,6], k: 30, aeq: 150 }
            - harmonic_torsion: { index: [5,6,7], k: 1, aeq: 110 }
            - harmonic_torsion: { index: [6,7,8], k: 30, aeq: 150 }
            - harmonic_torsion: { index: [7,8,9], k: 30, aeq: 150 }
        excluded_neighbours: 2
        keeppos: True
        rotate: False

insertmolecules:
    - intimin: {N: 1}

energy:
    - bonded: {}
    - nonbonded:
        default:
            - wca:
                mixing: LB 

moves:
    - transrot: {molecule: intimin, repeat: 8}
        
analysis:
    - savestate: {file: state.json}
    - savestate: {file: confout.pqr}
    - savestate: {file: confout.gro}
    - reactioncoordinate: {file: x.dat, nstep: 200, type: atom, property: x, index: 9}
    - reactioncoordinate: {file: y.dat, nstep: 200, type: atom, property: y, index: 9}
    - reactioncoordinate: {file: z.dat, nstep: 200, type: atom, property: z, index: 9}
    - xtcfile: {file: traj.xtc, nstep: 200}
    - sanity: {nstep: 10000}""")
```

Specify the path of the Faunus directory

In [ ]:

```
faunus_path = workdir+'/faunus'
```

### Run Monte Carlo simulation with a rigid D00-D0¶

In [ ]:

```
%%time
%cd $workdir
RT = 8.3145*.298
T = 298
!mkdir rigid
%cd rigid
with open('input.yml', 'w') as input_file:
    input_file.write(template.render(T=T, L=400, micro=8e6, k=30, aeq=150))
!{faunus_path}/scripts/yason.py input.yml > input.json
!{faunus_path}/faunus --input input.json --output output.json --nobar 
%cd ..
```

### Run Monte Carlo simulation with a flexible D00-D0¶

In [ ]:

```
%%time
!mkdir flexible50
%cd flexible50
with open('input.yml', 'w') as input_file:
    input_file.write(template.render(T=T, L=400, micro=8e6, k=2, aeq=110))
!{faunus_path}/scripts/yason.py input.yml > input.json
!{faunus_path}/faunus --input input.json --output output.json --nobar 
%cd ..
```

In [ ]:

```
!mkdir data
```

### Plot probability distributions of the domain–domain angle and the domain–linker separation¶

In [ ]:

```
model = 'rigid'
traj = md.load_xtc(model+'/traj.xtc',model+'/confout.gro')[100000:]
print(traj.n_frames)
r = md.compute_angles(traj,[[1,2,3]])/np.pi*180
edges = np.arange(r.min(),r.max(),1)
h, bins = np.histogram(r, bins=edges, density=True)
bins = bins[:-1] + (bins[1]-bins[0])/2.
np.savetxt('data/hist_cg_d00d1_rigid.dat',np.c_[bins,h])

model = 'flexible'
traj = md.load_xtc(model+'/traj.xtc',model+'/confout.gro')[100000:]
r = md.compute_angles(traj,[[1,2,3]])/np.pi*180
edges = np.arange(r.min(),r.max(),1)
h, bins = np.histogram(r, bins=edges, density=True)
bins = bins[:-1] + (bins[1]-bins[0])/2.
np.savetxt('data/hist_cg_d0d1_flexible.dat',np.c_[bins,h])

model = 'flexible'
r = md.compute_distances(traj,[[1,2]])
edges = np.arange(r.min(),r.max(),.01)
h, bins = np.histogram(r, bins=edges, density=True)
bins = bins[:-1] + (bins[1]-bins[0])/2.
np.savetxt('data/hist_cg_d00_flexible.dat',np.c_[bins,h])

model = 'flexible'
r = md.compute_distances(traj,[[2,3]])
edges = np.arange(r.min(),r.max(),.01)
h, bins = np.histogram(r, bins=edges, density=True)
bins = bins[:-1] + (bins[1]-bins[0])/2.
np.savetxt('data/hist_cg_d0_flexible.dat',np.c_[bins,h])
```

In [2]:

```
fig, (ax1,ax2) = plt.subplots(1, 2, sharex=False, sharey=False, figsize=(10,3.5))

bins,h = np.loadtxt('data/hist_cg_d00d1_rigid.dat',unpack=True)
ax1.plot(bins,h*1e2,color=plt.cm.tab10(0),lw=3,label='Rigid (CG)',ls='-')

bins,h = np.loadtxt('data/hist_cg_d0d1_flexible.dat',unpack=True)
ax1.plot(bins,h*1e2,color=plt.cm.tab10(3),lw=3,label='Flexible (CG)',ls='--')

###################################################

bins,h = np.loadtxt('data/hist_cg_d00_flexible.dat',unpack=True)
ax2.plot(bins,h,color=plt.cm.tab10(3),lw=3,label='D00-L1 (CG)',ls='-')

bins,h = np.loadtxt('data/hist_cg_d0_flexible.dat',unpack=True)
ax2.plot(bins,h,color=plt.cm.tab10(3),lw=3,label='D*-L* (CG)',ls='--')

ax1.set_ylabel('Probability x 10$^2$')
ax1.set_xlabel(r'Domain–Domain Angle, $\theta$  /  $\degree$')#,labelpad=10)

ax2.set_ylabel('Probability')
ax2.set_xlabel('Domain–Linker Separation  /  nm')#,labelpad=10)

ax2.yaxis.set_label_position("right")
ax2.yaxis.set_ticks_position('right')

ax1.annotate('a',xy=(.07,.85),fontsize=22,xycoords='axes fraction')
ax2.annotate('b',xy=(.07,.85),fontsize=22,xycoords='axes fraction')

ax1.legend(frameon=False,title='\n',handlelength=2.6)
ax2.legend(frameon=False,title='\n',handlelength=2.6)

plt.tight_layout()
plt.show()
```

### Plot 1D and 2D probability distributions of the angle w.r.t. the normal and the D3–D00 separation¶

In [5]:

```
def hist(model,color,label):
    x = np.loadtxt(model+'/x.dat',usecols=1)[100000:]/10. 
    y = np.loadtxt(model+'/y.dat',usecols=1)[100000:]/10. 
    z = np.loadtxt(model+'/z.dat',usecols=1)[100000:]/10.+15
    r = np.sqrt(x**2+y**2+z**2)
    alpha = np.arccos(z/r)/np.pi*180
    edgesx = np.arange(3,18,.5)
    Ex = edgesx[:-1]+(edgesx[1]-edgesx[0])/2.
    edgesy = np.arange(0,150,5)
    Ey = edgesy[:-1]+(edgesy[1]-edgesy[0])/2.
    
    h, _ = np.histogram(r, bins=edgesx, density=True)
    ax1.plot(Ex,h*10,color=color,lw=2,label=label)
    np.savetxt('figs/hist_dist_{:s}.dat'.format(model),np.c_[Ex,h])
    
    h, _ = np.histogram(alpha, bins=edgesy, density=True)
    ax2.plot(Ey,h*100,color=color)
    np.savetxt('figs/hist_angle_{:s}.dat'.format(model),np.c_[Ey,h])
    
def prob(ax,model,color):
    x = np.loadtxt(model+'/x.dat',usecols=1)[100000:]/10. 
    y = np.loadtxt(model+'/y.dat',usecols=1)[100000:]/10. 
    z = np.loadtxt(model+'/z.dat',usecols=1)[100000:]/10.+15
    r = np.sqrt(x**2+y**2+z**2)
    alpha = np.arccos(z/r)/np.pi*180
    edgesx = np.arange(3,18,.5)
    Ex = edgesx[:-1]+(edgesx[1]-edgesx[0])/2.
    edgesy = np.arange(0,150,5)
    Ey = edgesy[:-1]+(edgesy[1]-edgesy[0])/2.
    
    h, _, _ = np.histogram2d(alpha,r,bins=[edgesy,edgesx], density=True)

    im = ax.contourf(Ex, Ey, h*1000, 10, cmap=color)
    np.savetxt('figs/hist_2d_{:s}_Ex.dat'.format(model),Ex)
    np.savetxt('figs/hist_2d_{:s}_Ey.dat'.format(model),Ey)
    np.savetxt('figs/hist_2d_{:s}.dat'.format(model),h)
    return im
```

In [3]:

```
plt.rcParams['axes.labelsize'] = 12

ax0 = plt.subplot2grid(shape=(2,3), loc=(0,0), colspan=1, rowspan=2, fig=plt.figure(figsize=(10,6)))
ax1 = plt.subplot2grid((2,3), (0,1))
ax2 = plt.subplot2grid((2,3), (0,2))
ax3 = plt.subplot2grid((2,3), (1,1))
ax4 = plt.subplot2grid((2,3), (1,2))

ax0.axis('off')

model = 'rigid'
bins,h = np.loadtxt('data/hist_dist_{:s}.dat'.format(model),unpack=True)
ax1.plot(bins,h*10,color=plt.cm.tab10(0),lw=3)
bins,h = np.loadtxt('data/hist_angle_{:s}.dat'.format(model),unpack=True)
ax2.plot(bins,h*1e2,color=plt.cm.tab10(0),lw=3,label='Rigid\nD00-D0')


model = 'flexible'
bins,h = np.loadtxt('data/hist_dist_{:s}.dat'.format(model),unpack=True)
ax1.plot(bins,h*10,color=plt.cm.tab10(3),lw=3)
bins,h = np.loadtxt('data/hist_angle_{:s}.dat'.format(model),unpack=True)
ax2.plot(bins,h*1e2,color=plt.cm.tab10(3),lw=3,label='Flexible\nD00-D0')
ax2.set_xlim(0,150)
ax1.set_xlim(3,17.4)
ax1.set_ylabel(r'Probability x 10')
ax1.set_xlabel(r'D3–D00 Distance, $r$  /  nm',labelpad=10)
ax2.set_ylabel(r'Probability x 10$^2$')
ax2.set_xlabel(r'Angle w.r.t. Normal, $\alpha$  /  $\degree$',labelpad=10)


ax2.yaxis.set_label_position("right")
ax2.yaxis.set_ticks_position('right')

model = 'rigid'
Ex = np.loadtxt('data/hist_2d_{:s}_Ex.dat'.format(model))
Ey = np.loadtxt('data/hist_2d_{:s}_Ey.dat'.format(model))
h = np.loadtxt('data/hist_2d_{:s}.dat'.format(model))
im = ax3.contourf(Ex, Ey, h*1e3, cmap=plt.cm.Blues)
cbar = fig.colorbar(im,ax=ax3,label='Probability x 10$^3$',orientation='horizontal',pad=.3)
cbar.ax.set_xticklabels(['{:g}'.format(t) for t in np.arange(0,2.1,.25)])

model = 'flexible'    
Ex = np.loadtxt('data/hist_2d_{:s}_Ex.dat'.format(model))
Ey = np.loadtxt('data/hist_2d_{:s}_Ey.dat'.format(model))
h = np.loadtxt('data/hist_2d_{:s}.dat'.format(model))
im = ax4.contourf(Ex, Ey, h*1e3, cmap=plt.cm.Reds)
cbar = fig.colorbar(im,ax=ax4,label='Probability x 10$^3$',orientation='horizontal',pad=.3,)
cbar.ax.set_xticklabels(['{:g}'.format(t) for t in np.arange(0,2.1,.25)])
ax3.set_ylim(0,146)
ax4.set_ylim(0,146)
ax3.set_xlim(3,17.4)
ax4.set_xlim(3,17.4)
ax3.set_yticks([0,30,60,90,120])
ax4.set_yticks([0,30,60,90,120])

ax3.vlines(15.1,0,146,lw=.5,ls=':')
ax4.vlines(15.1,0,146,lw=.5,ls=':')

ax3.set_xlabel(r'D3–D00 Distance, $r$  /  nm')
ax3.set_ylabel(r'Angle w.r.t. Normal, $\alpha$  /  $\degree$',labelpad=1)

ax4.set_xlabel(r'D3–D00 Distance, $r$  /  nm')
ax4.set_ylabel(r'Angle w.r.t. Normal, $\alpha$  /  $\degree$',labelpad=1)

ax4.yaxis.set_label_position("right")
ax4.yaxis.set_ticks_position('right')

ax0.annotate('a',xy=(.15,.98),fontsize=22,xycoords='axes fraction')

for ax,l in zip([ax1,ax2],['b','c']):
    ax.annotate(l,xy=(.87,.84),fontsize=22,xycoords='axes fraction')
for ax,l in zip([ax3,ax4],['d','e']):
    ax.annotate(l,xy=(.87,.73),fontsize=22,xycoords='axes fraction')
ax2.legend(frameon=False,loc='center')

plt.tight_layout(h_pad = -1, w_pad = 0)
```

## Jacobian Correction¶

The histograms are divided by the square of the distance and the sine of the angle to remove geometric entropy contributions.
The probability distributions are normalized according to the following equations:

$$
\int\_0^\infty \mathrm{d}r P(r) r^2 = 1
$$$$
\int\_0^{180^\circ} \mathrm{d}\alpha P(\alpha) \sin{\alpha} = 1
$$$$
\int\_0^\infty \int\_0^{180^\circ} \mathrm{d}r \mathrm{d}\alpha P(r,\alpha) r^2 \sin{\alpha} = 1
$$

In [4]:

```
plt.rcParams['axes.labelsize'] = 12

ax0 = plt.subplot2grid(shape=(2,3), loc=(0,0), colspan=1, rowspan=2, fig=plt.figure(figsize=(10,6)))
ax1 = plt.subplot2grid((2,3), (0,1))
ax2 = plt.subplot2grid((2,3), (0,2))
ax3 = plt.subplot2grid((2,3), (1,1))
ax4 = plt.subplot2grid((2,3), (1,2))

ax0.axis('off')

model = 'rigid'
bins,h = np.loadtxt('data/hist_dist_{:s}.dat'.format(model),unpack=True)
h /= bins**2 # Jacobian correction
ax1.plot(bins,h*1e3,color=plt.cm.tab10(0),lw=3)
bins,h = np.loadtxt('data/hist_angle_{:s}.dat'.format(model),unpack=True)
h /= np.sin(bins/180*np.pi) # Jacobian correction
ax2.plot(bins,h*1e3,color=plt.cm.tab10(0),lw=3,label='Rigid\nD00-D0')


model = 'flexible'
bins,h = np.loadtxt('data/hist_dist_{:s}.dat'.format(model),unpack=True)
h /= bins**2 # Jacobian correction
ax1.plot(bins,h*1e3,color=plt.cm.tab10(3),lw=3)
bins,h = np.loadtxt('data/hist_angle_{:s}.dat'.format(model),unpack=True)
h /= np.sin(bins/180*np.pi) # Jacobian correction
ax2.plot(bins,h*1e3,color=plt.cm.tab10(3),lw=3,label='Flexible\nD00-D0')
ax2.set_xlim(0,150)
ax1.set_xlim(3,17.4)
ax1.set_ylabel(r'Probability x 10$^3$')
ax1.set_xlabel(r'D3–D00 Distance, $r$  /  nm',labelpad=10)
ax2.set_ylabel(r'Probability x 10$^3$')
ax2.set_xlabel(r'Angle w.r.t. Normal, $\alpha$  /  $\degree$',labelpad=10)


ax2.yaxis.set_label_position("right")
ax2.yaxis.set_ticks_position('right')

model = 'rigid'
Ex = np.loadtxt('data/hist_2d_{:s}_Ex.dat'.format(model))
Ey = np.loadtxt('data/hist_2d_{:s}_Ey.dat'.format(model))
h = np.loadtxt('data/hist_2d_{:s}.dat'.format(model))
h /= (Ex*Ex*np.sin(Ey/180*np.pi).reshape(-1,1)) # Jacobian correction
im = ax3.contourf(Ex, Ey, h*1e5, cmap=plt.cm.Blues)
cbar = fig.colorbar(im,ax=ax3,label='Probability x 10$^5$',orientation='horizontal',pad=.3)
cbar.ax.set_xticklabels(['{:g}'.format(t) for t in np.arange(0,2.1,.25)])

model = 'flexible'    
Ex = np.loadtxt('data/hist_2d_{:s}_Ex.dat'.format(model))
Ey = np.loadtxt('data/hist_2d_{:s}_Ey.dat'.format(model))
h = np.loadtxt('data/hist_2d_{:s}.dat'.format(model))
h /= (Ex*Ex*np.sin(Ey/180*np.pi).reshape(-1,1)) # Jacobian correction
im = ax4.contourf(Ex, Ey, h*1e5, cmap=plt.cm.Reds)
cbar = fig.colorbar(im,ax=ax4,label='Probability x 10$^5$',orientation='horizontal',pad=.3,)
cbar.ax.set_xticklabels(['{:g}'.format(t) for t in np.arange(0,2.9,.4)])
ax3.set_ylim(0,146)
ax4.set_ylim(0,146)
ax3.set_xlim(3,17.4)
ax4.set_xlim(3,17.4)
ax3.set_yticks([0,30,60,90,120])
ax4.set_yticks([0,30,60,90,120])

ax3.vlines(15.1,0,146,lw=.5,ls=':')
ax4.vlines(15.1,0,146,lw=.5,ls=':')

ax3.set_xlabel(r'D3–D00 Distance, $r$  /  nm')
ax3.set_ylabel(r'Angle w.r.t. Normal, $\alpha$  /  $\degree$',labelpad=1)

ax4.set_xlabel(r'D3–D00 Distance, $r$  /  nm')
ax4.set_ylabel(r'Angle w.r.t. Normal, $\alpha$  /  $\degree$',labelpad=1)

ax4.yaxis.set_label_position("right")
ax4.yaxis.set_ticks_position('right')

ax0.annotate('a',xy=(.15,.98),fontsize=22,xycoords='axes fraction')

for ax,l in zip([ax1,ax2],['b','c']):
    ax.annotate(l,xy=(.87,.84),fontsize=22,xycoords='axes fraction')
for ax,l in zip([ax3,ax4],['d','e']):
    ax.annotate(l,xy=(.87,.73),fontsize=22,xycoords='axes fraction')
ax2.legend(frameon=False,loc='center left')

plt.tight_layout(h_pad = -1, w_pad = 0)
```
