# Supplementary material for: The extracellular juncture domains in the intimin passenger adopt a constitutively extended conformation inducing restraints to its sphere of action
Source: Sci Rep. 2020 Dec 4;10:21249. doi: 10.1038/s41598-020-77706-7 (PMC7718877; doi:10.1038/s41598-020-77706-7)
Supplement: Supplementary file 5 — Supplementary Information 3. [file 41598_2020_77706_MOESM5_ESM.docx]

**The extracellular juncture domains in the intimin passenger adopt a constitutively extended conformation inducing restraints to its sphere of action**

Julia Weikum,^1,2^ Alina Kulakova,^3^ Giulio Tesei,^5^ Shogo Yoshimoto,^6^ Line Vejby Jægerum,^1^ Monika Schütz,^7^ Katsutoshi Hori,^6^ Marie Skepö,^8^ Pernille Harris,^3,4^ Jack C. Leo,^9,10*^ and J. Preben Morth^1,2,11^*

^1^ Membrane Transport Group, Centre for Molecular Medicine Norway (NCMM), Nordic EMBL Partnership, University of Oslo, P.O. Box 1137 Blindern, 0318 Oslo, Norway

^2^ Enzyme and Protein Chemistry, Section for Protein Chemistry and Enzyme Technology, Department of Biotechnology and Biomedicine, Technical University of Denmark, Søltofts Plads, 2800, Kgs. Lyngby, Denmark

^3^ Department of Chemistry, Technical University of Denmark, Kemitorvet building 207, 2800 Kgs. Lyngby, Denmark

^4^ Department of Chemistry, University of Copenhagen, Universitetsparken 5, DK-2100 Copenhagen, Denmark.

^5^ Structural Biology and NMR Laboratory & the Linderstrøm-Lang Centre for Protein Science, Department of Biology, University of Copenhagen, Copenhagen N, Denmark

^6^ Department of Biomolecular Engineering, Graduate School of Engineering, Nagoya University, Furo-cho, Chikusa-ku, Nagoya 464-8603, Japan

^7^ Interfaculty Institute for Microbiology and Infection Medicine, Institute for Medical Microbiology and Hygiene, University Hospital Tübingen, 72076 Tübingen, Germany

^8^ Division of Theoretical Chemistry, Department of Chemistry, Lund University, 221 00 Lund, Sweden

^9^ Department of Biosciences, University of Oslo, P.O. Box 1137 Blindern, 0318 Oslo, Norway.

^10^ Department of Biosciences, Nottingham Trent University, Nottingham NG11 8NS, UK

^11^ Institute for Experimental Medical Research (IEMR), Oslo University Hospital, Ullevål PB 4956 Nydalen, NO-0424 Oslo, Norway.

*Correspondence: jack.leo@ntu.ac.uk, premo@dtu.dk

Short title: Structural and *in silico* characterization of the intimin passenger

**Supplementary figures**

**Figure S1**

**
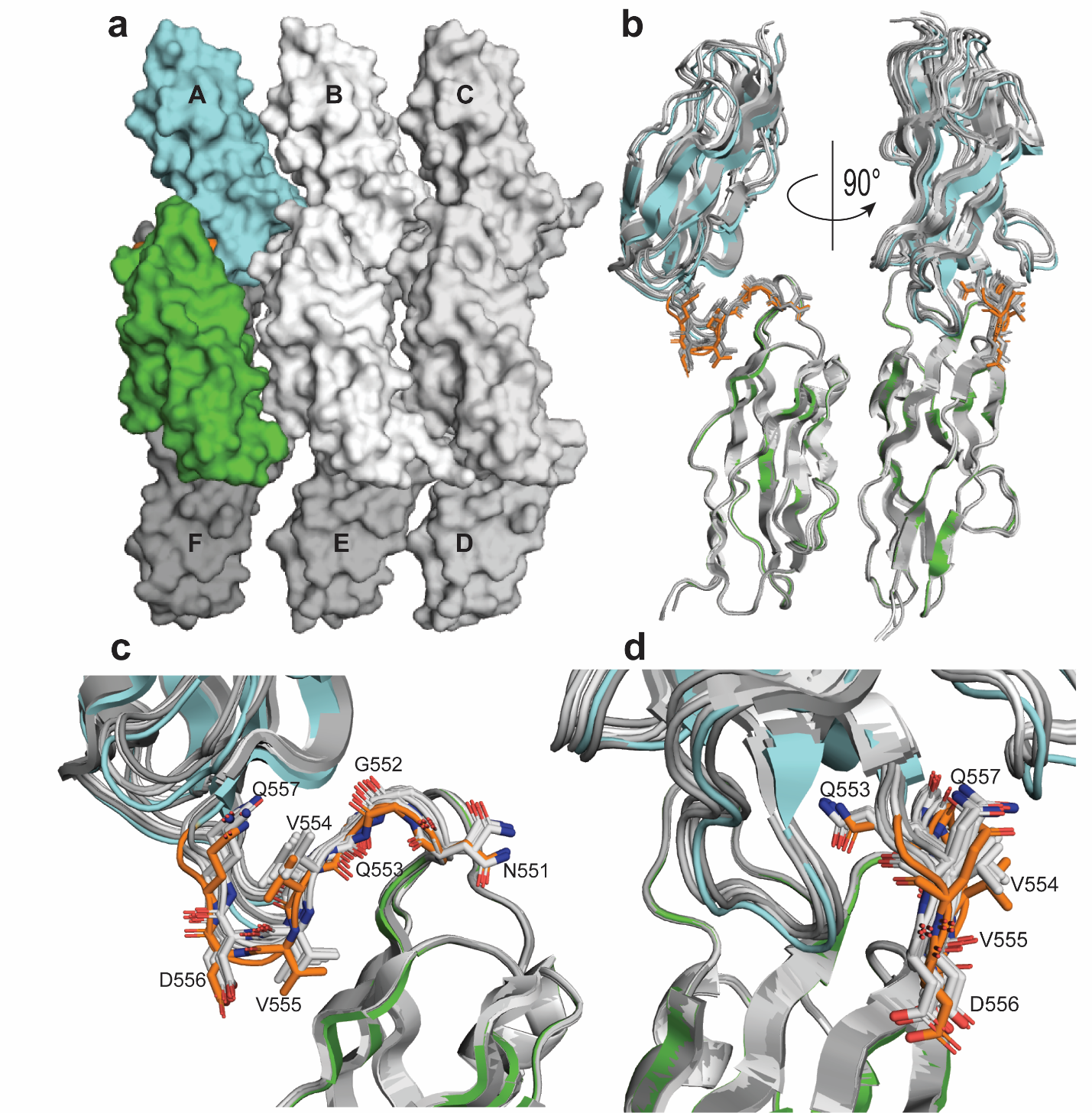
**

**Figure S2
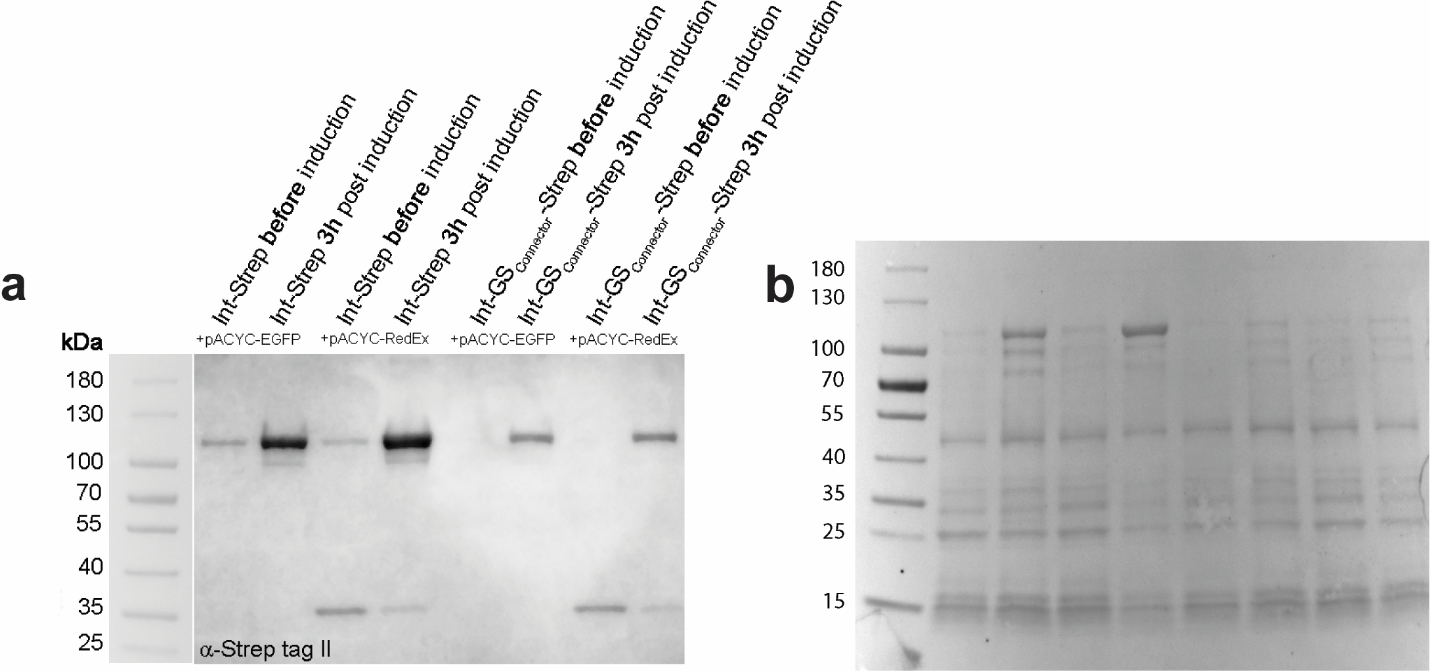
**

**Figure S3**

**
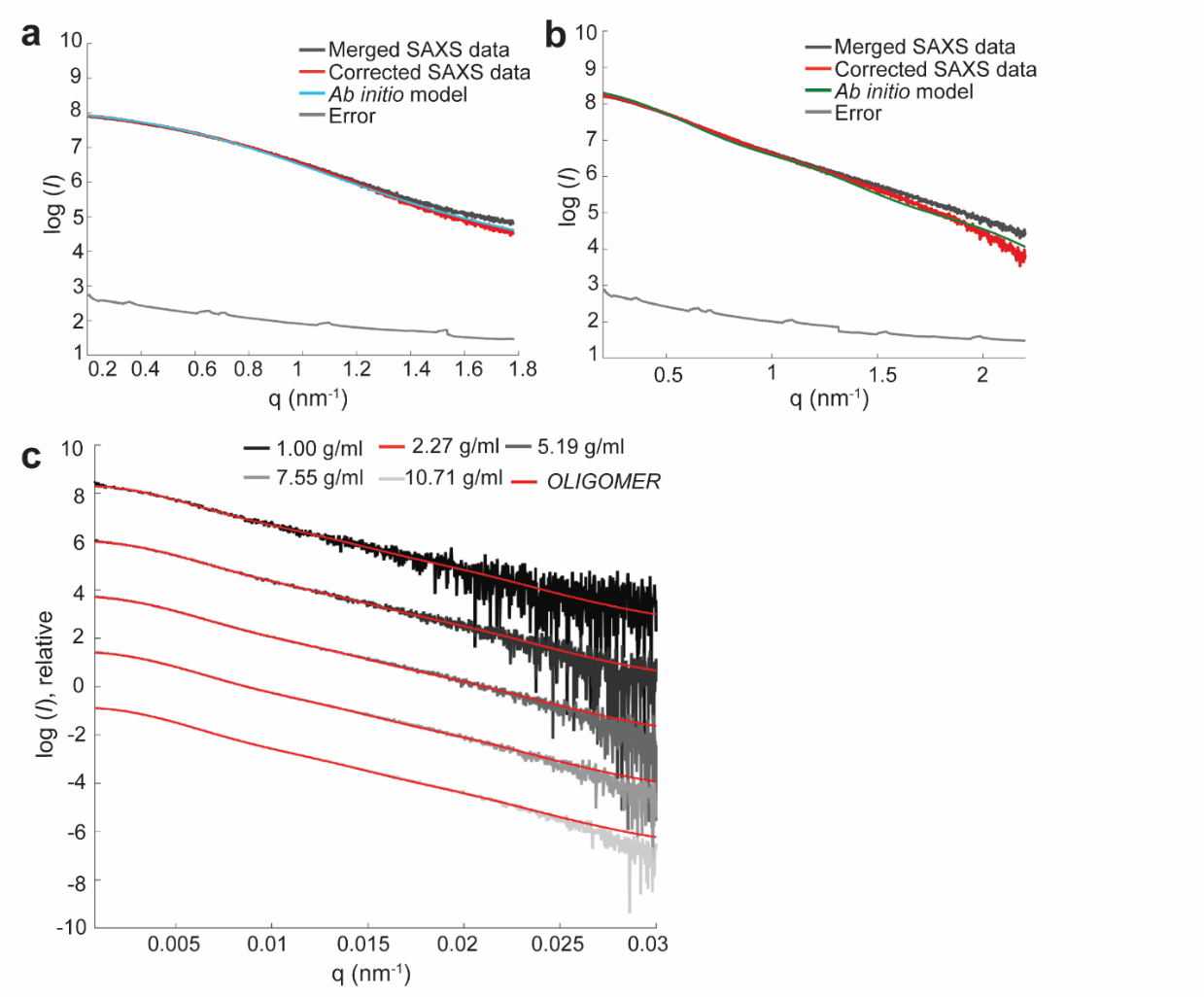
**

**Figure S4**

**
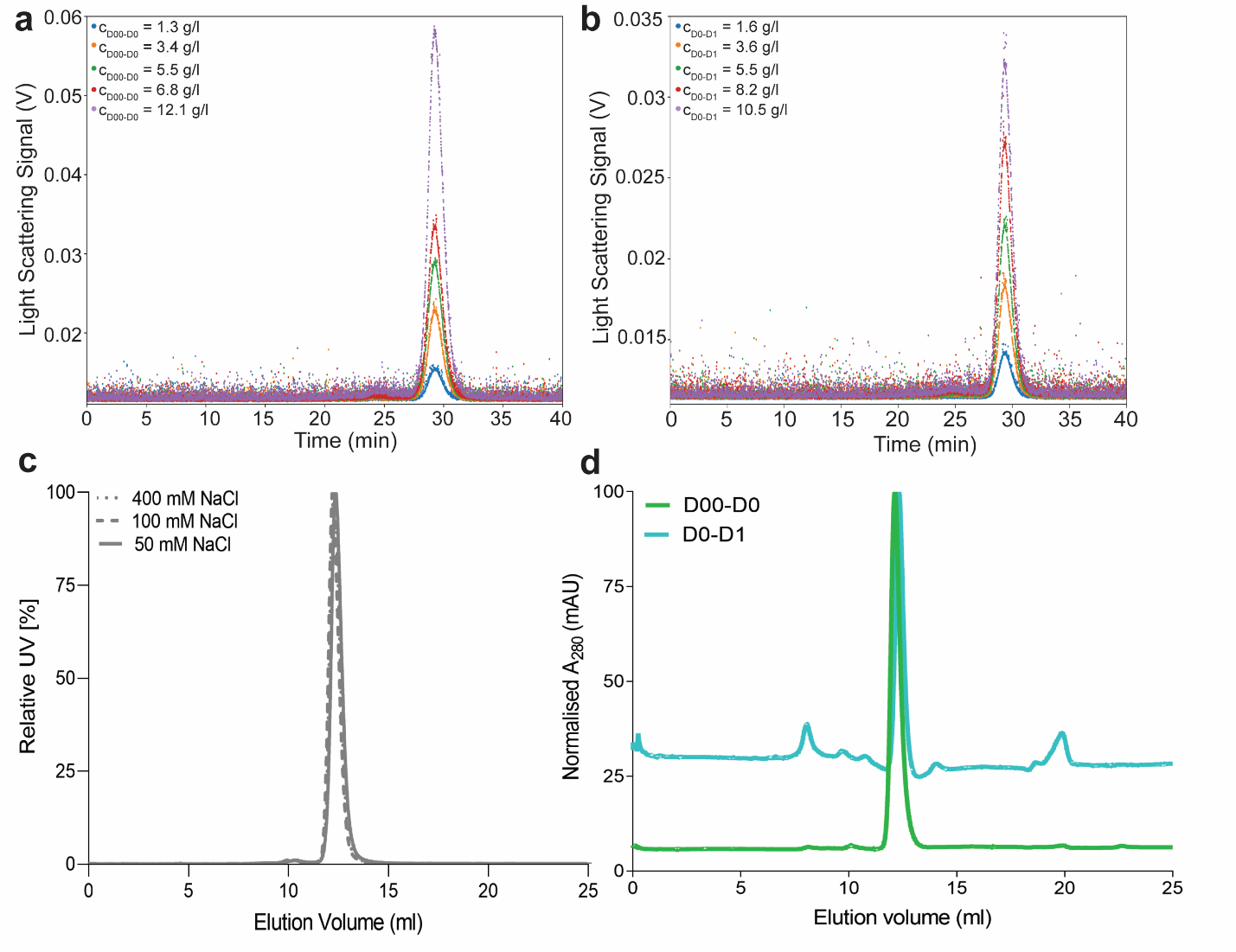
**

**Figure S5**

**
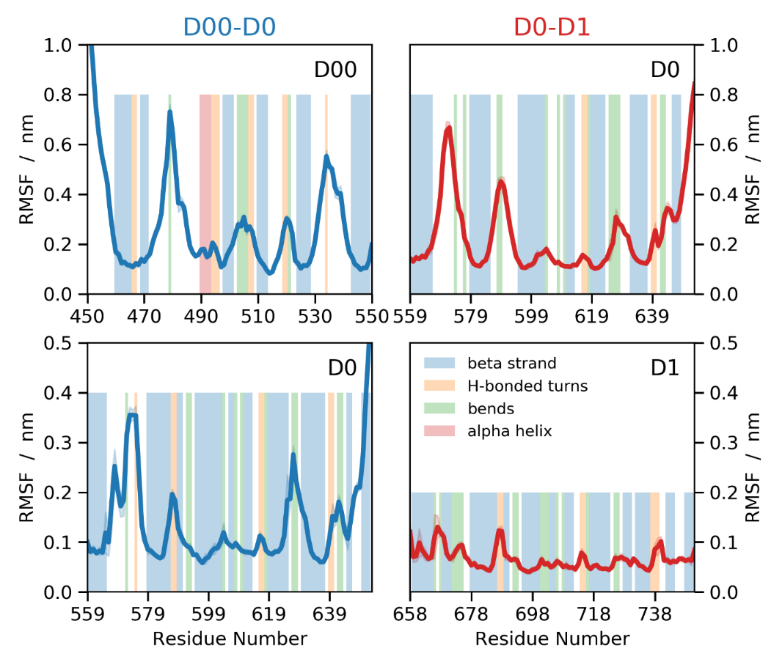
**

**Figure S6**

**
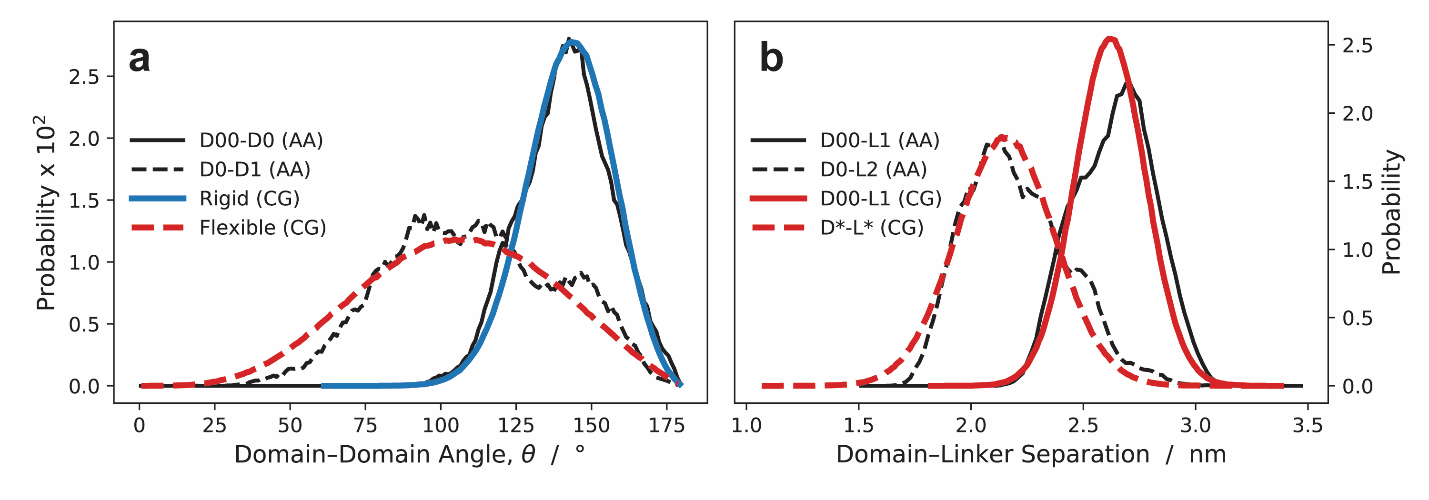
**

**Figure S7**

**
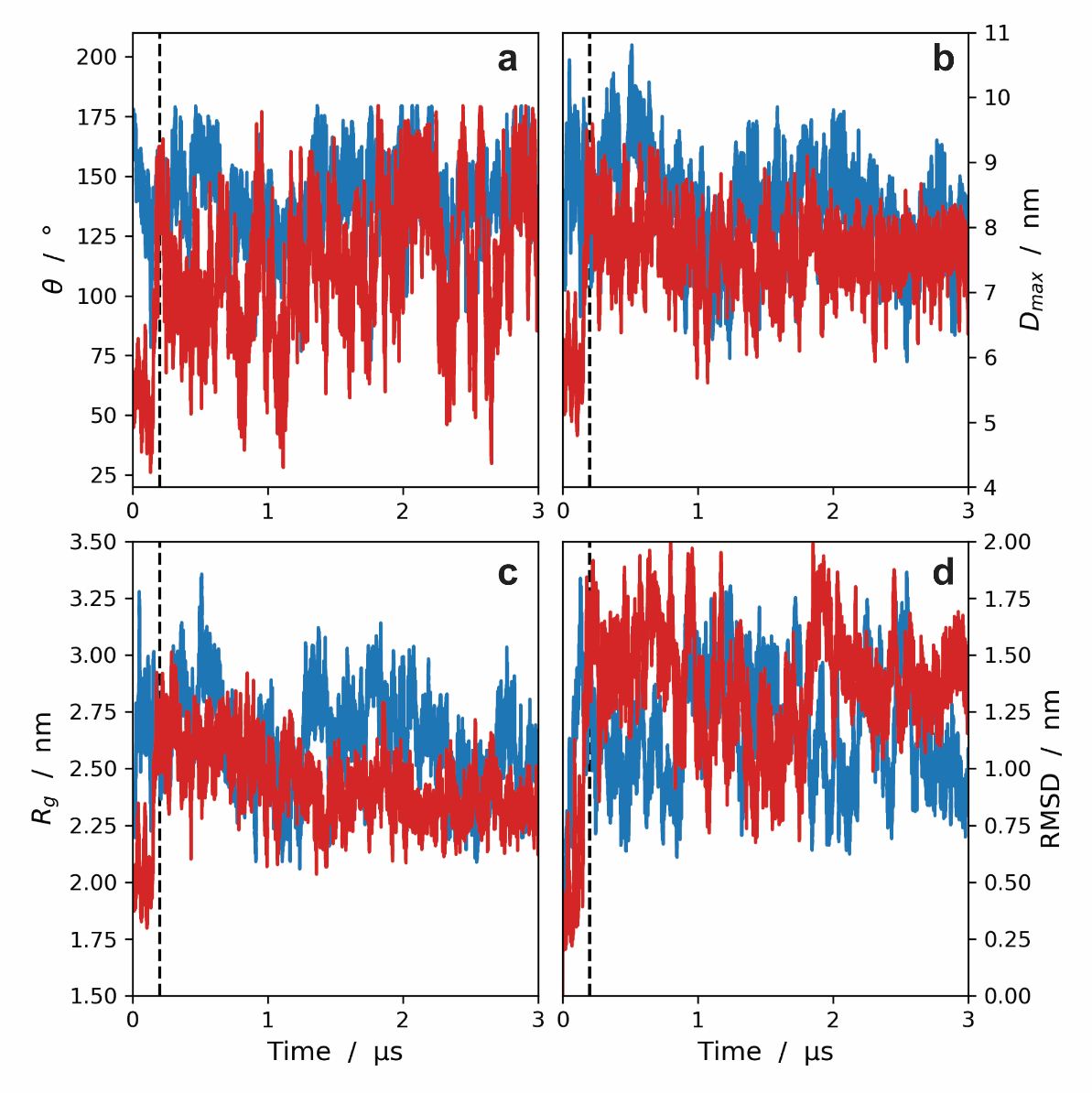
**

**Supplementary figure legends**

**Figure S1: Superimposition of individual D00-D0 chains in the asymmetric unit**

(a) Asymmetric unit of D00-D0 containing six chains as marked. Chain A is represented in the color scheme as shown in Figure 1. Chain B-F are represented in gray scale. (b) Superimposition of six D00-D0 chains of the asymmetric unit. Molecules were superimposed in Pymol to chain A residue 454-549, corresponding to D00. Color scheme corresponds to a. (c, d) Close-up of superimposition on D00-D0 linker region. Molecular graphics were presented with PyMOL 2.2.7 Molecular Graphics System (Schrödinger, LLC).

**Figure S2: Expression of intimin constructs Int-Strep and Int-GS_Connector_-Strep**

(a) Whole cell lysates corresponding to 2.5 x 10^6^ cells per lane were analyzed by SDS-PAGE and western blot. Int-Strep and Int-GS_Connector_-Strep were detected in samples harvested before and after 3 h of induction with AHTC using an anti-Strep tag II antibody. For both Intimin variants clones were tested that additionally harboured either pACYC-EGFP or pACYC-RedEx.

(b) Ponceau staining of western blot shows equal loading for all samples.

**Figure S3: *Ab initio* modeling and concentration dependent *OLIGOMER* fit on SAXS scattering of D0-D1 and D00-D0**

(a) Fitting curve (cyan) of *ab initio* model to merged and corrected SAXS data of D0-D1

(χ^2^ = 2.9).

(b) Fitting curve (green) of *ab initio* model to merged and corrected SAXS data of D00-D0

(χ^2^ = 18.5).

(c) Fitting curves (red) of *OLIGOMER* analysis on scattering curves of D00-D0 at different protein concentrations. *OLIGOMER* was run against models of *EOM* analysis, protein crystal structure and dimer formation based on crystals structure.

**Figure S4: Absolute mass determination of D00-D0 and D0-D1**

(a) SEC-MALS profiles of increasing concentrations of (a) D00-D0 and (b) D0-D1 on Superdex75 Increase 10/300 GL (GE). The condition and concentrations correspond to experimental settings used for SAXS experiments described in Figure 3. Parameters derived from SEC-MALS analysis are presented in Table S8.

(c) Analytical size exclusion profile of subdomains D00-D0 on Superdex75 Increase 10/300 GL (GE) in the presence of increasing sodium chloride concentration. Analytical SEC revealed no salt-dependence on D00-D0. D00-D0 is present as a monomer in all conditions. (d) Analytical size exclusion profile of subdomains D00-D0 (green) and D0-D1 (cyan) on Superdex75 Increase 10/300 GL (GE). Molecular weight determined by analytical SEC of D00-D0 (28.4 kDa) (dark green) and D0-D1 (26.2 kDa) (cyan) exceeds the theoretical molecular weight of both proteins.

**Figure S5: Intimin subdomains vary in domain rigidity**

Root-mean-square fluctuation (RMSF) of each residue in the D00 (K450–S550), D0 (G559–V653), and D1 (T658–F751) domains of D00-D0 (*left panels*) and D0-D1 (*right panels*). Higher RMSF indicates increased flexibility. Shaded areas indicate the secondary structure assignment into β-strands (blue), H-bonded turns (orange), bends (green), and α-helices (red).

**Figure S6: Comparison between intradomain probability distributions obtained from the all-atom and the coarse-grained model**.

(a) Probability distributions of the domain-domain angles in the all-atom (AA) simulations of D00-D0 (black solid line) and D0-D1 (black dashed line). The colored lines are the D00-D0 angle distributions obtained from the coarse-grained (CG) model using the flexible (red line) and the rigid (blue line) harmonic potential. (b) Probability distributions of the mass-center separations between the connector region L1 and the domains D00 (solid lines) and D0 (dashed lines). Black and red lines are calculated from AA and CG simulations, respectively.

**Figure S7: Time-resolved implicit solvent molecular dynamics simulations of D00-D0 and D0-D1**

Time evolution of (a) the domain-domain angle, $\theta$; (b) the maximum dimension, $D_{max}$; (c) the radius of gyration,$R_{g};$ (d) the RMSD with respect to the crystal structure of D00-D1 (blue) and D0-D1 (red) calculated from implicit solvent all-atom MD simulations. Dashed black vertical lines indicate the last trajectory frame excluded from the analysis of the simulation data.

**Supplementary Tables**

**S1 Table: Primer sequences used in cloning**

| **Primers** | **Sequences** |
| --- | --- |
| D00-D0 | **For**: CGA GGG CAA AAA ATG CAT CAC CAT CAC CAT CAC AAG CAG GAT ATT CTT TCT CTG AAT ATT  **Rev**: CAC AGG TCA AGC TTA TTA TTG ATC AAC AAA TAT AAC TGC ATT GG |
| D0-D1 | **For**: AAC CTG TAT TTT CAG AGC AAT GGT CAG GTG GTC GAC CA  **Rev**: CAC AGG TCA AGC TTA TTA TGT AAA AAA TTC AAC TTC AGG TGC TTT |
| pASK-IBA3 | **For**: TAA TAA GCT TGA CCT GTG AAG TGA  **Rev**: CAT TTT TTG CCC TCG TTA TCT AGA T |
| TEV insertion | **For**: CCTGTATTTTCAGAG C AAG CAG GAT ATT CTT TCT CTG AAT ATT  **Rev**: CTC TGA AAA TAC AGG TTTTC GTG ATG GTG ATG GTG ATG CAT |
| pIBA3-His-TEV | **For**: TAA TAA GCT TGA CCT GTG AAG TGA  **Rev**: CTC TGA AAA TAC AGG ttttc GTG ATG GTG ATG GTG ATG CAT |
| GS_connector_ | **For**: GGT TCT GGC AGT GGT AGC GGC GTT GGG GTA ACG GAC TTT ACG  **Rev**: CAC TGC CAG AAC C CGA CAG AAC GGT AAT AGT AAG CA |
| pTac-promoter | **For:** GCG AAG CTT GCC AGT GTG CTG GAA TTC G  Rev: CCG GAT CCC CGG GAA TTC GTA ATC ATG GA |
| EGFP | **For:** CGC GGA TCC ATG GTG AGC AAG GGC GAG G  **Rev:** GGG TCG ACT TAC TTG TAC AGC TCG TCC ATG C |
| RedEx | **For:** CGC GGA TCC ATG GCC TCC TCC GAG GAC G  **Rev:** GGG TCG ACT TAC AGG AAC AGG TGG TGG CGG C |

**S2 Table: SAXS – experimental details**

| a) Sample details | | | |
| --- | --- | --- | --- |
|  | | D00-D0 | D0-D1 |
| Organism | | *E. coli* O127:H6 E2348/69 (EPEC)  Taxonomy ID: 574521 | *E. coli* O127:H6 E2348/69 (EPEC)  Taxonomy ID: 574521 |
| Extinction coefficient with His-tag (A_280_, M^-1^cm^-1^) | | 15930 | 5960 |
| Molecular mass *M* from chemical composition with His-tag (kDa) | | 22.9 | 22.6 |
| b) SAXS data collection | | | |
| Instrument | P12 BioSAXS beamline (PETRAIII) | | |
| Date | 12^th^ July 2019 | | |
| Detector | Pilatus6m | | |
| Wavelength (nm) | 0.123981 | | |
| Beam size (mm^2^) | 0.2 × 0.12 | | |
| Detector distance (m) | 4.0 | | |
| *q-*measurement range (nm^-1^) | 0.017- 5.506 | | |
| Absolute scaling method | Comparison with scattering from BSA | | |
| Normalization | To transmitted intensity by beam-stop counter | | |
| Monitoring for radiation damage | Frame-by-frame comparison | | |
| Exposure time (s) | 20 x 0.195 | | |
| Sample configuration | Quartz glass capillary | | |
| Sample temperature (ºC) | 20 | | |
| c) Software employed for SAXS data reduction, analysis and interpretation | | | |
| SAS data reduction | *PRIMUSqt* (Konarev *et al.*, 2003) from *ATSAS* 2.8.3 (Franke *et al.*, 2017) | | |
| Extinction coefficient estimate | *ExPaSy* (Gasteiger *et al.*, 2003) | | |
| Basic analyses: Guinier, *p*(*r*), *V*_P_ | *PRIMUSqt* (Konarev *et al.*, 2003) | | |
| *Ab initio* modelling | *GASBOR* (Svergun, Petoukhov and Koch, 2001) | | |

**S3 Table: Structural parameters derived from SAXS experiments**

|  | *c*_protein_ (g/L) | Guinier | | *p*(*r*) | | | Porod Volume  (nm^3^) | Apparent *MW* (kDa) | | |
| --- | --- | --- | --- | --- | --- | --- | --- | --- | --- | --- |
|  |  | *I*(0)/*c* | *R*_g_  (nm) | *I*(0)/*c* | *R*_g_ (nm) | *D*_max_ (nm) |  | Guinier | *p*(*r*) | Porod Volume |
| D00-D0 | 1.0 | 3972 | 2.7 | 3967 | 2.8 | 8.8 | 31 | 24.6 | 24.6 | 18.0 |
|  | 2.3 | 3940 | 2.7 | 3944 | 2.8 | 8.8 | 32 | 24.4 | 24.5 | 18.9 |
|  | 5.3 | 4011 | 2.8 | 4012 | 2.8 | 9.5 | 32 | 24.9 | 24.9 | 19.0 |
|  | 7.7 | 4051 | 2.8 | 4051 | 2.9 | 9.5 | 33 | 25.1 | 25.1 | 19.2 |
|  | 11.0 | 4110 | 2.8 | 4126 | 2.9 | 9.8 | 33 | 25.5 | 25.6 | 19.5 |
| D0-D1 | 0.8 | 3962 | 2.1 | 3916 | 2.1 | 5.8 | 30 | 24.6 | 24.3 | 17.7 |
|  | 1.9 | 4017 | 2.2 | 3940 | 2.1 | 5.8 | 31 | 24.9 | 24.4 | 18.1 |
|  | 4.2 | 3982 | 2.2 | 3918 | 2.1 | 5.8 | 30 | 24.7 | 24.3 | 17.9 |
|  | 6.0 | 3993 | 2.2 | 3909 | 2.1 | 5.8 | 30 | 24.8 | 24.3 | 17.8 |
|  | 8.9 | 4002 | 2.2 | 3909 | 2.1 | 5.8 | 30 | 24.8 | 24.3 | 17.6 |

**S4 Table: Superimposition of D00-D0 chain A to chain B-F, respectively, and the root mean square deviation**

| **D00-D0 chain A superimposed to** | **RMSD (Å)** |
| --- | --- |
| D00-D0 chain B | 0.15 |
| D00-D0 chain C | 0.25 |
| D00-D0 chain D | 0.2 |
| D00-D0 chain E | 0.16 |
| D00-D0 chain F | 0.18 |

**S5 Table: Contour length increments and force peaks derived from AFM experiments**

|  | | **Int-WT_connector_** | **Int-GS_connector_** |
| --- | --- | --- | --- |
| Number of measurements | | 40 | 47 |
| Unfolding event 1 | |  |  |
|  | Δ*L_C_* (nm) | 31.2 ± 2.4 | 30.8 ± 1.9 |
|  | Max. Force (pN) | 244.7 ± 45.2 | 256.1 ± 39.9 |
| Unfolding event 2 | |  |  |
|  | Δ*L_C_* (nm) | 30.2 ± 2.4 | 30.9 ± 1.9 |
|  | Max. Force (pN) | 292.9 ± 48.4 | 283.5 ± 31.9 |
| Unfolding event 3 | |  |  |
|  | Δ*L_C_* (nm) | 32.2 ± 2.0 | 31.9 ± 1.8 |
|  | Max. Force (pN) | 315.9 ± 28.4 | 305.9 ± 43.9 |

**S6 Table: Fit parameters of D0-D1 with *EOM***

| Model | *R*_g_ (nm) | *D*_max_ (nm) | Volume fraction | ꭓ2 |
| --- | --- | --- | --- | --- |
| Bent | 2.015 | 6.125 | 0.808 | 1.708 |
| Extended | 2.870 | 9.859 | 0.202 |  |

**S7 Table: Fit parameters of D00-D0 with *OLIGOMER***

|  | *c*_protein_ (g/L) | Bent | Extended | Dimer | ꭓ2 |
| --- | --- | --- | --- | --- | --- |
| D00-D0 | 1.00 | 0.281 (±0.016) | 0.536 (±0.016) | 0.184 (±0.005) | 1.04 |
|  | 2.27 | 0.233 (±0.007) | 0.561 (±0.007) | 0.205 (±0.002) | 1.47 |
|  | 5.19 | 0.270 (±0.004) | 0.518 (±0.004) | 0.212  (±0.001) | 2.91 |
|  | 7.55 | 0.295 (±0.003) | 0.478 (±0.003) | 0.227 (±0.001) | 3.65 |
|  | 10.71 | 0.330 (±0.003) | 0.424 (±0.003) | 0.246 (±0.001) | 6.80 |

**S8 Table: Parameters derived from SEC-MALS experiments**

|  | *c*_protein_ (g/L) | MW (kDa) | Error % |
| --- | --- | --- | --- |
| D00-D0 | 1.28 | 22.63 | 2.845 |
|  | 3.43 | 22.98 | 1.006 |
|  | 5.52 | 22.42 | 0.523 |
|  | 6.76 | 22.84 | 0.655 |
|  | 12.07 | 22.25 | 0.456 |
| D0-D1 | 1.56 | 22.53 | 1.744 |
|  | 3.61 | 22.41 | 0.818 |
|  | 5.53 | 22.40 | 0.763 |
|  | 8.15 | 22.05 | 0.641 |
|  | 10.5 | 22.16 | 0.517 |

**Supporting Data files**

Supplementary Data 1, Weikum_data_1_coarse-grained_model.ipynb

Supplementary Data 2, Weikum_Data_2_coarse-grained_model.html

Also available on GitHub at https://github.com/gitesei/SI-intimin.
